# Nanoparticle-based Cell Trackers for Biomedical Applications

**DOI:** 10.7150/thno.39915

**Published:** 2020-01-12

**Authors:** Jen-Shyang Ni, Yaxi Li, Wentong Yue, Bin Liu, Kai Li

**Affiliations:** 1 Department of Biomedical Engineering, Academy for Advanced Interdisciplinary Studies, Southern University of Science and Technology, Shenzhen, Guangdong 518055, China; 2 HKUST-Shenzhen Research Institute, Shenzhen 518057, China; 3 School of Life Science and Technology, Harbin Institute of Technology, Harbin 150080, China; 4 Department of Chemical and Biomolecular Engineering, National University of Singapore, Singapore

**Keywords:** aggregation-induced emission, bioimaging, cell tracking, semiconducting polymer, therapy

## Abstract

The continuous or real-time tracking of biological processes using biocompatible contrast agents over a certain period of time is vital for precise diagnosis and treatment, such as monitoring tissue regeneration after stem cell transplantation, understanding the genesis, development, invasion and metastasis of cancer and so on. The rationally designed nanoparticles, including aggregation-induced emission (AIE) dots, inorganic quantum dots (QDs), nanodiamonds, superparamagnetic iron oxide nanoparticles (SPIONs), and semiconducting polymer nanoparticles (SPNs), have been explored to meet this urgent need. In this review, the development and application of these nanoparticle-based cell trackers for a variety of imaging technologies, including fluorescence imaging, photoacoustic imaging, magnetic resonance imaging, magnetic particle imaging, positron emission tomography and single photon emission computing tomography are discussed in detail. Moreover, the further therapeutic treatments using multi-functional trackers endowed with photodynamic and photothermal modalities are also introduced to provide a comprehensive perspective in this promising research field.

## Introduction

Continuous *in vivo* cell tracking over a long period of time can offer valuable information in detail regarding cellular processes and biomedical therapies 1-3. A variety of biomedical imaging techniques, including positron emission tomography (PET) 4-6, single photon emission computing tomography (SPECT) 7, magnetic resonance imaging (MRI) 1,8-10, magnetic particle imaging (MPI) 11-13, photoacoustic (PA) imaging 14-18 and fluorescence imaging 19-25, have been explored for such applications from bench side to bedside 3. As such, the invention of versatile contrast agents as long-term cell trackers to monitor the target at least over several weeks is of high importance in translational research. Currently, two major categories of cell labeling strategies, direct labeling and indirect labeling, have been implemented in practice. Each strategy has its own advantages and disadvantages. In general, direct labeling approach enjoys the advantages of easy preparation, high labeling efficiency, and abundant availability of exogenous contrast agents, while indirect labeling strategy involving genetic modification can afford permanent cell tagging. Among them, bioluminescence, a natural light source based on luciferase catalysis oxidation of its luciferin substrate, is a typical and most well-adapted indirect labeling technology.

**Figure 1:**
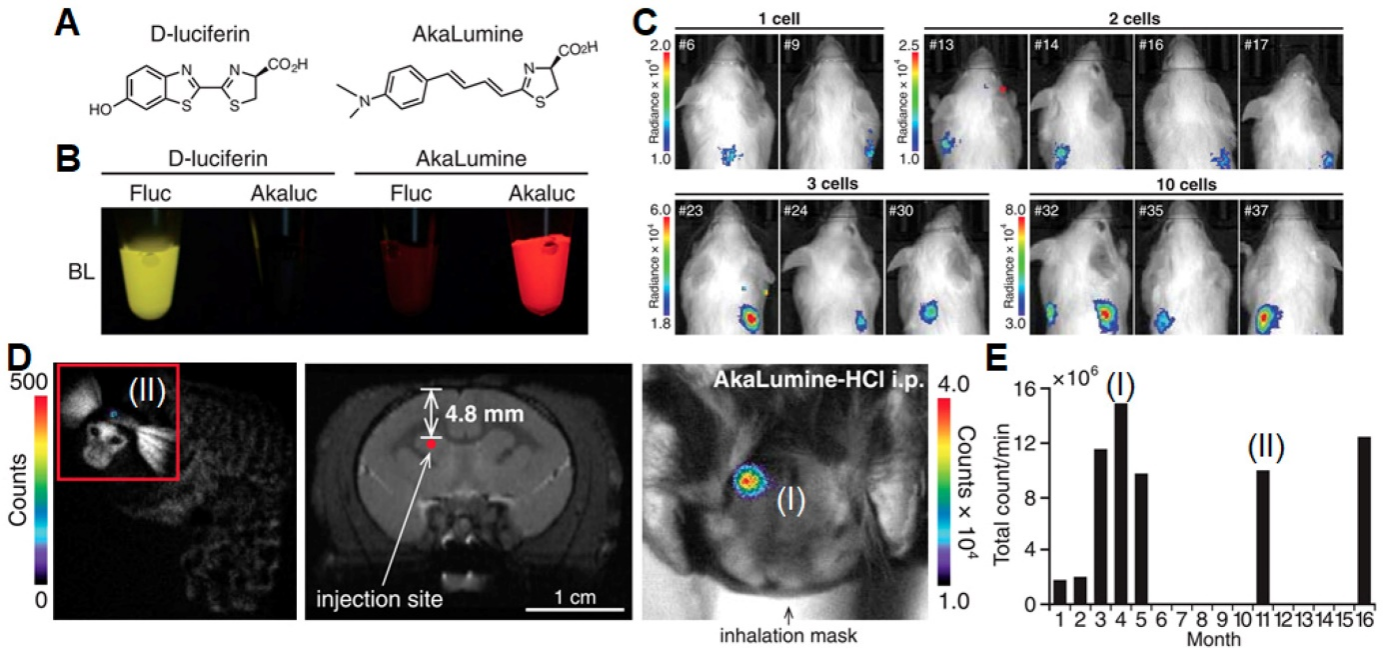
(A) Chemical structures of D-luciferin and AkaLumine. (B) Bioluminescence imaging of four mixtures of substrate (100 mM) and enzyme (2 mg mL^‒1^; Fluc: firefly luciferase; Akaluc, screened from Fluc-based library). (C) Analysis of single-cell and sparse-cell AkaBLI of implanted tumorigenic cells trapped in mouse lung. (D) Chronic video-rate AkaBLI of brain striatal neurons in a common marmoset. (E) Quantified bioluminescence signals against time after injection. Reprinted with permission from 27, copyright 2018 American Association for the Advancement of Science.

Luciferase catalyzes the oxidization of luciferin by intramolecular oxygen, leading to oxyluciferin molecule in the excited state. After emitting in the excited state, the molecule reduces back to luciferin substrate. This technique has shown promising potentials in a wide range of *in vitro* and *in vivo* applications, including immunoassays, gene expression analyses, drug screening, bioimaging of living systems, as well as diagnosis and microenvironmental monitoring of tumors 26. Bioluminescence does not need external light irradiation, which helps avoid interference from background fluorescence and biological auto-fluorescence signals during imaging. Thus, bioluminescence-based methods are extremely sensitive to provide good spatial resolution in a wide dynamic range. Inspired by the unique property of bioluminescence, Miyawaki *et al.* designed a bioluminescence imaging system (named AkaBLI) that produces *in vivo* emission signals 100 to 1000-fold brighter as compared with conventional technology (**Figure 1**) 27. They recorded video-rate bioluminescent signals from neurons in the striatum, a deep brain area, for more than a year. This study indicates that the red-emissive and highly deliverable luciferin analog (AkaBLI) can serve as a bioengineered light source to motivate unidentified scientific, medical, and engineering applications. Advances in bioluminescence imaging methods allowed researchers to measure tumor growth, visualize growing processes, and track cell-cell interactions 28,29.

Nevertheless, many challenges and limitations still exist in bioluminescence imaging technology. For example, the imaging requires highly sensitive CCD lens and unstable bioluminescence suffers from signal decay. In addition, long detection time due to their weak signals, high cost owing to the repeated luciferin injection from time to time, and the risk of transgenic markers transfecting on cells, genes, or antibodies are all of major concerns that impede their progress in translational research. On the other hand, green fluorescent protein (GFP) and its variants, another major category of genetic cell tagging in indirect labeling strategies, are restricted by their poor photostability, inherent susceptibility to enzymes and interference from bio-substrate autofluorescence 30,31. Alternatively, exploration of exogenous contrast agents, such as nanoparticle (NP)-based cell trackers, for biomedical and/or preclinical investigation has attracted a broad research interest. For now, several nanoparticles, including superparamagnetic iron oxide NPs (SPIONs), carbon NPs, silver NPs and CdS/ZnS quantum dots, have been tested in the clinical trials (**Table 1**). Therein, SPIONs for MRI-guiding pancreatic cancer and silver NPs-based central venous catheters for catheter related infections have been completed in phase 4 clinical trials. This indicates that the preclinical development and exploration of NP-based trackers or agents are of great importance. In this review, we thus mainly focus on the recent research and application of NP-based direct labeling strategy for long-term cell-tracking of biomedical progress. Their potential theranostic applications are also briefly discussed to inspire the future exploration of multi-functional cell trackers.

## Nanoparticles for long-term cell tracking

### Cell tracking *via* magnetic resonance imaging (MRI)

Magnetic resonance imaging (MRI) offers much higher spatial resolution (50 μm) than bioluminescence imaging, which allows *in vivo* cell tracking combined with detailed anatomical information of individual organs 1. To date, iron oxide NPs (IONs) 32,33, gadolinium (Gd)-based NPs 34, manganese (Mn)-based NPs 6,35, ^19^F-based NPs 8,36,37 and SPIONs 13,38-41 have served as MRI contrast agents to show promising results for* in vivo* cell tracking. These contrast agents have exhibited good biocompatibility and effectiveness, high spatial resolution, desired penetration depth and non-ionizing radiation 42.

**Figure 2:**
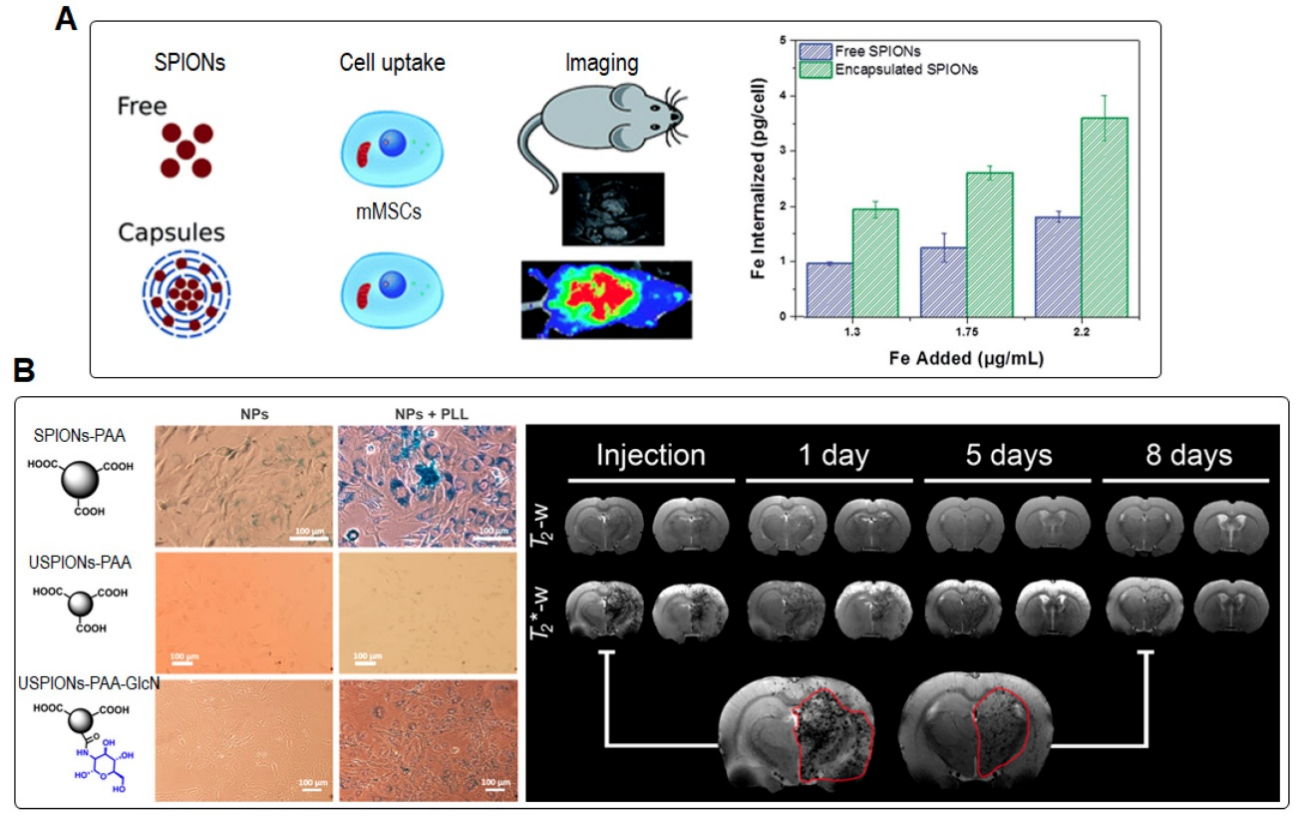
(A) Diagram of labeling mouse bone marrow derived mesenchymal stem cells (mMSCs) with free and encapsulated SPIONs, and internalization of free and encapsulated SPIONs versus the feeding concentration for cell labeling, determined from ferrozine assay. Reprinted with permission from 39, copyright 2019 Royal Society of Chemistry. (B) Prussian blue staining of MSCs incubated with SPIONs-PAA, USPIONs-PAA, and USPIONs-PAA-GlcN NPs, respectively, in the presence or absence of polylysine (PLL) at 100 μg Fe mL^-1^ over 24 h at 37 °C, and representative T_2_- and T_2_*-weighted MRI of a healthy rat injected intra-arterially with USPIONs-PAA-GlcN-labeled MSCs at different time points. Reprinted with permission from 2, copyright 2017 American Chemical Society.

Recently, Ashraf *et al.* labeled mouse bone marrow-derived mesenchymal stem cells (MSCs) with SPIONs encapsulated by polyelectrolyte multilayers. At a low feeding concentration (100 μg Fe mL^-1^), the encapsulated SPIONs served as an efficient labeling agent for stem cells, which had a 2-fold higher uptake efficiency of Fe in comparison with that labeled with bare SPIONs (**Figure 2A**) 39. The bio-distribution after intra-cardiac injection of labeled cells was monitored longitudinally by MRI. Through 2 weeks of *in vivo* tracking, the particles released from dead cells mainly accumulated in liver and spleen without complete clearance. To further enhance the cellular uptake and biocompatibility, Rivas *et al.* used glucosamine to modify polyacrylic acid (PAA)-coated ultrasmall iron oxide nanoparticles (USPIO-PAA-GlcN) 43 and label MSCs for tracking by MRI, while PAA-coated SPIONs (SPIONs-PAA) and PAA-coated USPIONs (USPIONs-PAA) were used for comparison (**Figure 2B**) 2. After incubation with MSCs, the SPIONs-PAA were only found in a portion of cells with nonuniform distribution due to their severe aggregation behavior in cell culture medium. On the other hand, USPIONs-PAA showed good *in vitro* colloidal stability but low intracellular internalization efficiency. By contrast, USPIO-PAA-GlcN displayed good biocompatibility, high cellular uptake (56.5 pg Fe/cell) and excellent sensitivity in both* in vitro* and *in vivo* MRI experiments, showing promises as a labeling agent for cell tracking applications in animal models of cerebral ischemia 44,45. Lately, Daldrup-Link *et al.* utilized ferumoxytol-labeled matrix-associated stem cell implants (MASIs) to predict cartilage repair outcomes with MRI in a minipig model. The ferumoxytol-labeled MASIs showed significant T2 shortening compared with unlabeled MASIs (22.2 msec ± 3.2 vs 27.9 msec ± 1.8; *P* < 0.001), facilitating MR imaging of the MASI upon transplantation in cartilage defects. In addition, ferumoxytol-labeled apoptotic MASIs showed a higher T2 relaxation time compared with the labeled viable MASIs 2 weeks post implantation (26.6 msec ± 4.9 vs 20.8 msec ± 5.3; *P* = 0.001) due to the rapid iron loss post cell death. Correspondingly, standard MRI showed incomplete cartilage defect repair in the apoptotic MASI-treated group at 24 weeks. These results suggested that the commercial ferumoxytol as MR trackers can facilitate early diagnosis of failed MASIs within 2 weeks in a large-animal model 46. In addition to single-modality imaging applications, MRI synergized with PET or fluorescence imaging modalities have great potentials for dual-/multi-modality tracking to provide more comprehensive biological information, especially in translational research and clinical practice 6,36.

### Cell tracking *via* magnetic particle imaging (MPI)

**Figure 3:**
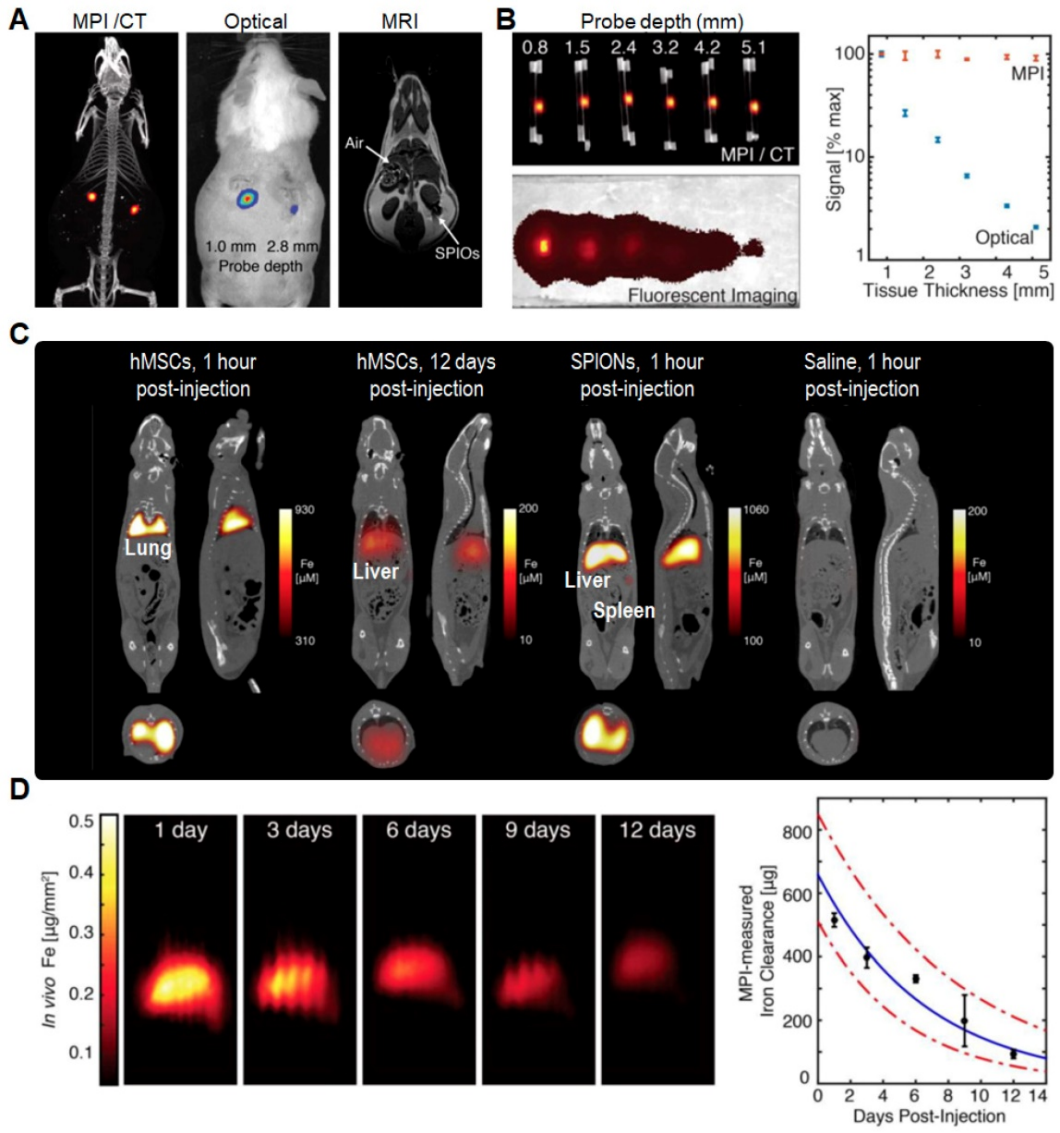
(A) Comparison of MPI/CT, fluorescence imaging, and MRI in mouse. (B) Quantitative comparison of MPI and fluorescent signal in different tissue depths. (C) MPI/CT imaging of mice intravenously injected with hMSCs, SPIONs, and saline. (D) MPI quantification of *in vivo* SPIONs clearance upon hMSCs injections. Reprinted with permission from 47, copyright 2016 Ivyspring.

Over the last decade, magnetic particle imaging (MPI) has been an emerging imaging approach with near-ideal image contrast, penetration depth and robustness to image IONs-labeled cells *in vivo*. Especially, it has shown ultra-sensitive and linearly quantitative imaging in practice. As compared to MRI, MPI only requires non-ionizing and low frequency magnetic fields that are safer for clinical translation. In 2016, Zheng *et al.* used MPI technique to dynamically monitor and quantify the biodistribution of human mesenchymal stem cells (hMSCs) labeled by SPIONs (**Figure 3**) 47. They first demonstrated that the MPI signal was not significantly dependent on surrounding tissue depth and hence can be clinically translatable for applications in molecular imaging (**Figure 3B**). After tail vein injection, the labeled hMSCs were immediately entrapped in lung tissues with the Fe concentration of ~52 ng Fe mm^‒3^, and then migrated to the liver within one day. In stark contrast, the standard SPIONs upon tail vein injection were directly taken up by liver and spleen (**Figure 3C**) 48,49. Through monitoring *in vivo* distribution and clearance of labeled hMSCs over a period of 12 days, they obtained a gradual decay of MPI signal in liver with a half-life of 4.6 days and 95% confidence intervals between 3.7 and 6.0 days (**Figure 3D**).

**Figure 4:**
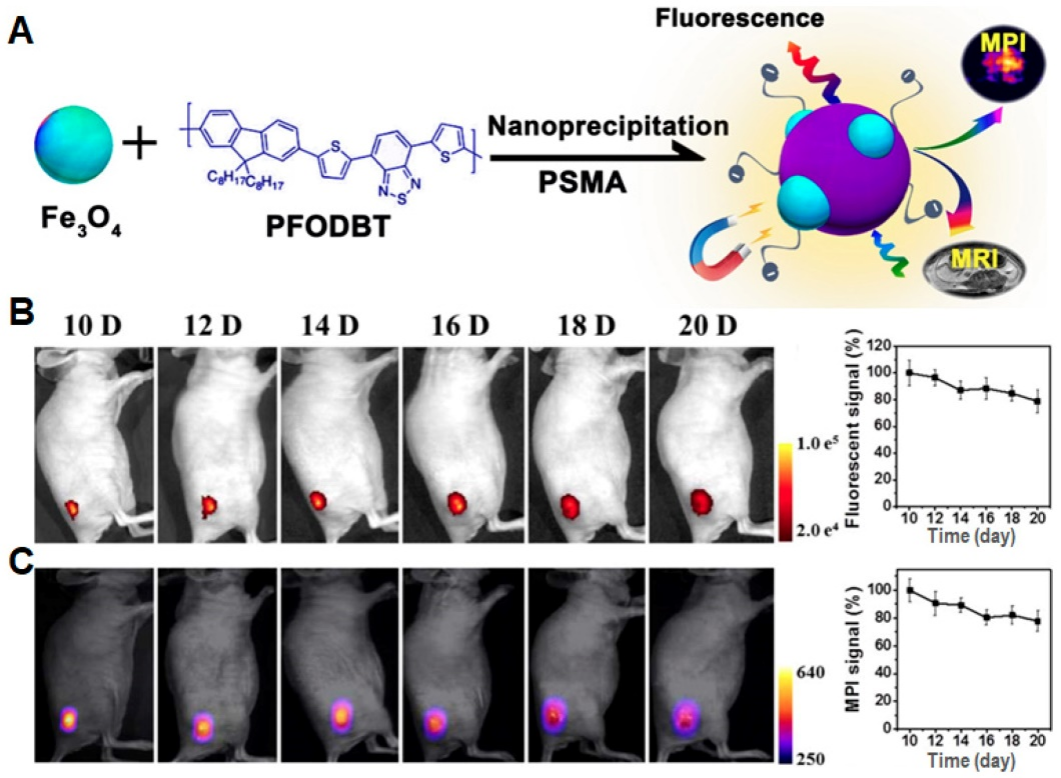
(A) Schematic illustration of the preparation of Janus nanoparticles through nanoprecipitation. (B) Longitudinal fluorescence images of a representative mouse at different time points (*λ*_ex_: 540 nm; *λ*_em_: 680 nm), and their quantification of fluorescence signals (%) for tumor areas versus post implantation time, normalized to the value of day 10 post-implantation. (C) Longitudinal two-dimensional projection MPI images of a representative mouse, and their quantification of MPI signals (%) for tumor areas versus post implantation time, normalized to the value of day 10 post-implantation. Reprinted with permission from 12, copyright 2018 American Chemical Society.

To further improve MPI performance, Rao *et al.* utilized a semiconducting polymer (SP; PFODBT) and IONs to produce Janus NPs with an average size of 27 nm in diameter, which showed both magnetic and optical properties in MPI and fluorescence imaging (**Figure 4A**) 12. At the same Fe concentration, MPI signal of the Janus NPs showed a 3-fold and 7-fold enhancement as compared to that of the commercial MPI tracker (Vivotrax) and MRI contrast agent (Feraheme), respectively. During *in vivo* tumor-growth tracking, fluorescence and MPI signals only decreased by 20% from day 10 to day 20 post-implantation (**Figure 4B and C**). These results indicate that MPI can allow imaging of the magnetic tracers in deep tissues with an outstanding linearity between tracer amount and signal intensity for quantitative detection. Recently, they developed a NIR-emissive Janus NPs (MMPF NPs) serving as magneto-optical multimodal nanoplatform to image xenografted tumors in living mice 50. MMPF NPs with an average diameter of 42 nm by TEM possessed a long-term blood circulation time (49 h of half-life) and high tumor uptake efficiency (18% ID/g), leading to high tumor to normal tissue contrast in subcutaneous and orthotopic tumor models. Interestingly, using MPI technique, they observed the disappeared NPs signal in the liver upon day 14 post injection and the visible signal in the spleen after day 85. Among the existing molecular imaging modalities, MPI shows a unique combination of high sensitivity, quantitative accuracy, and longitudinal monitoring in long-term cell tracking study. As such, the multi-modal technology combining MRI, PA and fluorescence imaging can simultaneously enhance the detecting and tracking accuracy, which will further promote the development of preclinical and/or clinical cell therapies 11,13.

### Cell tracking *via* fluorescence imaging

In comparison with the traditional fluorescent probes such as endogenous biomolecules and fluorescent proteins, fluorescent NPs exist unique advantages of higher brightness, tunable fluorescence and better photobleaching resistance 51. In this section, we focus on a series of fluorescent NPs 52 for cell tracking, including inorganic quantum dots (QDs) 53,54, nanodiamonds 55,56, and organic NPs 51,57. For organic NPs, we emphasize on aggregation-induced emission (AIE) dots 57-59 and semiconducting polymer nanoparticles (SPNs) 60-62 as cell trackers, resulting from their excellent photostability, long-term cell tracking ability, and good biocompatibility 63-65.

### Inorganic quantum dots: QDs

**Figure 5:**
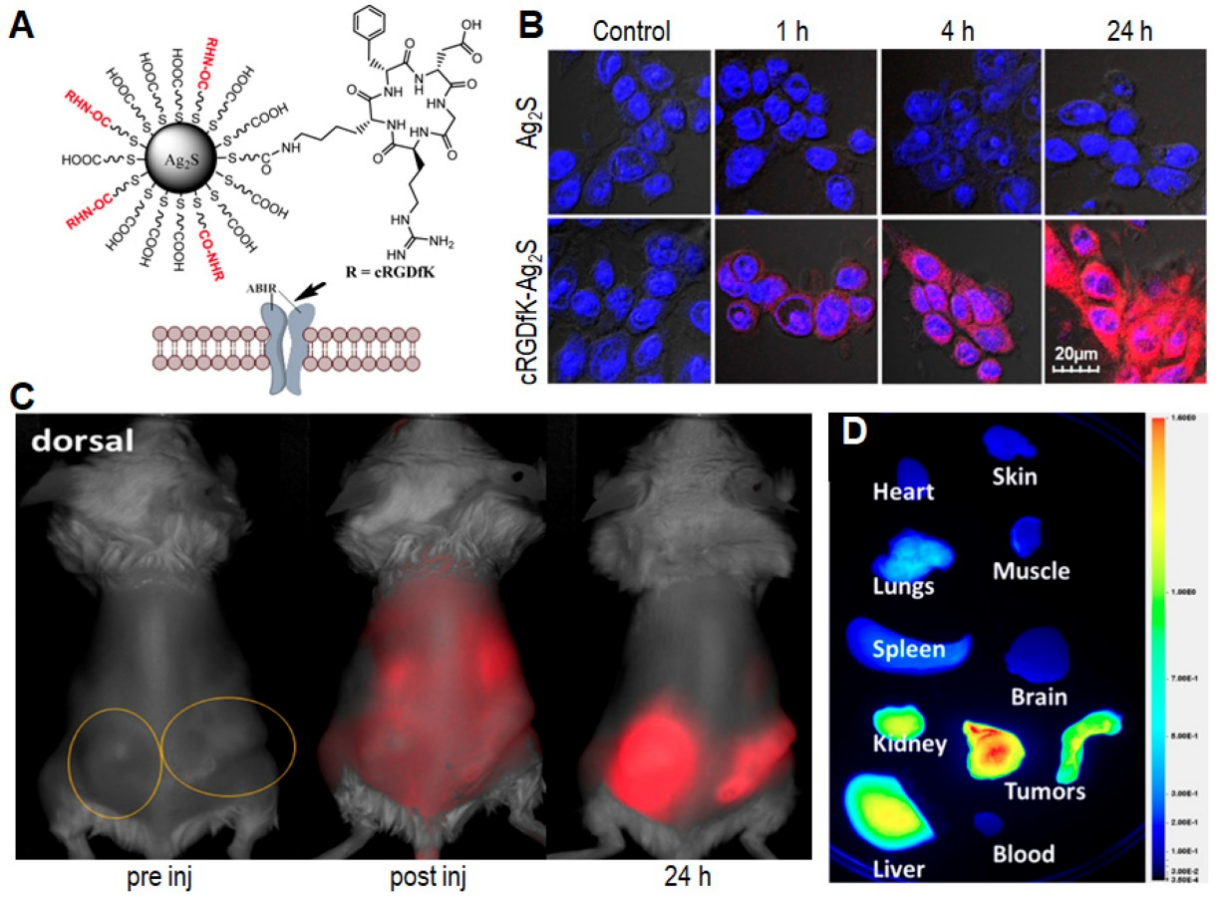
(A) Schematic illustration of the cRGDfK-Ag_2_S QDs. (B) Evaluation of internalization of QDs in 4T1luc cells at different time points. (C) Representative *in vivo* fluorescence imaging of cRGDfK-Ag_2_S in 4T1luc tumor-bearing Balb/c mouse after intravenous administration. The circles indicate bilateral subcutaneous tumor locations. (D) Representative *ex vivo* fluorescence image of organ tissues from 4T1luc bilateral tumor bearing mice 24 h post injection. Reprinted with permission from 86, copyright 2015 American Chemical Society.

Quantum dots (QDs) are a class of inorganic semiconductor nanocrystals with unique photophysical properties, including long fluorescence lifetime, high fluorescence quantum yield, and excellent photostability 66-68. However, traditional inorganic QDs have shown inherent limitations such as high toxicity and short circulation time 69. For instance, a study has reported that QDs could induce abnormalities during embryo development 70 or depolarize mitochondria membranes to cause potential cytotoxicity 71. In addition, QDs encapsulated by exotic polymer materials have been reported to cause inevitable immune response 72, while QDs modified by biomacromolecules such as proteins and genes led to drug tolerance and systematic toxicity 73. To address this critical issue, series of novel QDs, such as silver sulfide (Ag_2_S) 54,74-77, silver selenide (Ag_2_Se) 78, and lead sulfide (PbS) 79,80 QDs, have been explored for a variety of biomedical applications including cell tracking 53,81-83. Among these QDs, Ag_2_S QDs have been most successfully used for cell tracking due to their superior photostability and high quantum yields in the second near-infrared (NIR-II) window 53,66,84. Wang *et al.* encapsulated an Ag_2_S QD with fluorescence in the NIR-II window (1000-1700 nm) with protein nanocages (PNCs), using simian virus 40 (SV40) PNC (PNC_SV40_) as a model, to monitor the *in vivo* behaviors of PNCs 54,74. Benefiting from the high spatiotemporal resolution and deep tissue penetration of NIR-II fluorescence imaging 85, the dynamic *in vivo* distribution of PNC_SV40_ in living mice was tracked in a real time manner 54. Besides, they successfully used Ag_2_S QD-labeled hMSCs to continuously monitor the fluorescence signal in animals up to 30 days 76. The *in vivo* NIR-II imaging clearly revealed the heparin-facilitated translocation of hMSCs from lung to liver and the long-term retention of hMSCs in the liver for treatment of liver failure 76. Furthermore, they used Ag_2_S QDs to monitor lymphatic drainage and vascular networks with high spatial resolution, and track angiogenesis mediated by a tiny tumor *in vivo* 75. Soon afterwards, Achilefu *et al.* conjugated a tumor-avid cyclic pentapeptide (Arg-Gly-Asp-DPhe-Lys, cRGDfk) to ultra-small Ag_2_S QDs (named cRGDfK-Ag_2_S QDs; **Figure 5A**) with tunable light emission from 500 to 1200 nm and a hydrodynamic diameter below 10 nm 86. Compared with the non-conjugated ones, the water-soluble cRGDfK-Ag_2_S QDs showed selective integrin-mediated internalization in cancer cells (**Figure 5B**). From *in vivo* and *ex vivo* fluorescence images (**Figure 5C** and **D**), an exceptionally high tumor-to-liver uptake ratio can be observed 24 h post injection, indicating that cRGDfK-Ag_2_S QDs had high targeting ability to the α_v_β_3_ integrin receptors to promote their accumulation in tumor tissues *via* receptor-mediated endocytosis 87. As a result, such cRGDfK-Ag_2_S QDs provide an efficient approach to construct nanoplatforms that are able to selectively target disease biomarkers in living organisms 86,88.

In regenerative medicine, promoted differentiation of the transplanted stem cells can facilitate the overall healing progresses. To address this issue, Bain *et al.* developed a QD-based multifunctional NP (RGD-*β*-CD-QDs) with an average diameter of 4~5 nm 89. Its compact size facilitates the cellular uptake of QDs and further delivers the hydrophobic osteogenic dexamethasone and siRNA into the stem cells to enhance their osteogenesis differentiation. Moreover, the *in vivo* fluorescent signal of the RGD-*β*-CD-QDs labeled hMSCs could be observed on day 21 post implantation. These results clearly evidence that such functional QD-based trackers can provide a powerful tool to simultaneously enhance differentiation and long-term tracking of hMSCs* in vitro* and *in vivo*. Besides QDs, the same group also reported a multifunctional nanocarrier based on inorganic upconversion nanoparticles for controlled differentiation and long-term tracking of hMSCs. Upon exposure of NIR light, the emission in UV region leads to photocleavage of the photocaged linker and intracellular release of differentiation-inducing kartogenin, which further triggered chondrogenic differentiation of hMSCs for promoted neocartilage formation *in vivo* 90,91.

### Organic material-based nanoparticles

**Figure 6:**
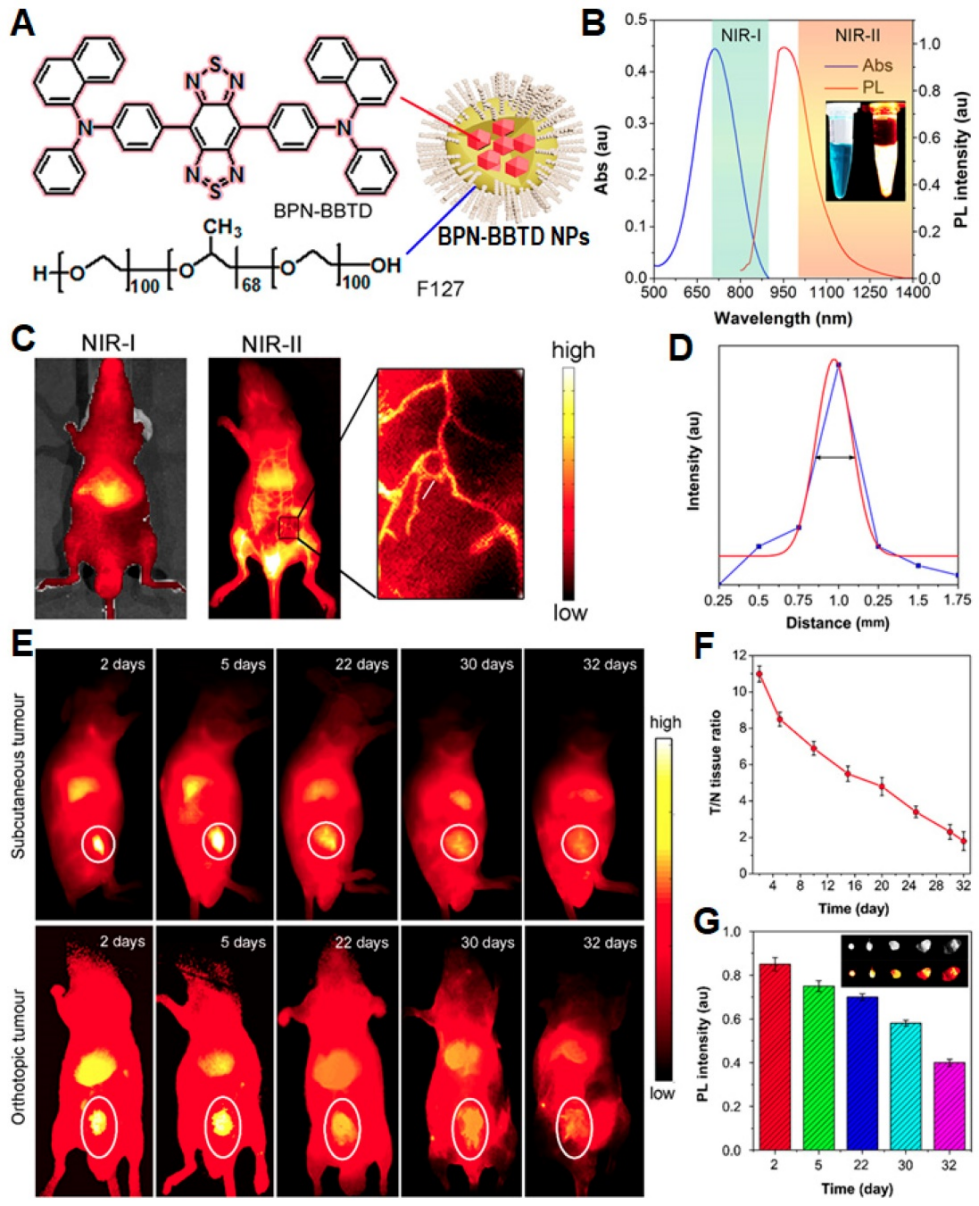
(A) Schematic illustration of BPN-BBTD NPs. (B) Absorption and PL spectra of BPN-BBTD NPs in aqueous dispersion. (C) NIR-I (*λ*_ex_: 700 nm) and NIR-II (*λ*_ex_: 785 nm) fluorescence images of the nude mice intravenously injected with aqueous dispersion of BPN-BBTD NPs 15 min post-treatment. (D) A cross-sectional fluorescence intensity profile (blue) along the white line in the treated mouse in the inset of panel (C). (E) Representative time-dependent *in vivo* NIR-II fluorescence images of subcutaneous and orthotopic bladder-tumor-bearing mice treated with BPN-BBTD NPs at a dose of 1 mg mL^-1^ (200 μL; *λ*_ex_: 785 nm, 10 mW cm^-2^). (F) Time-dependent ratios of NIR-II fluorescence intensity from circled tumor site in panel (E) to that from normal tissue (T/N) in subcutaneous bladder-tumor-bearing mice. (G) Time-dependent normalized NIR-II fluorescence intensities and images (inset) of tumors extracted from the orthotopic bladder-tumor-bearing mice. Reprinted with permission from 105, copyright 2018 American Chemical Society.

As compared to QDs, organic material-based NPs with nontoxic luminogens are potential alternatives for* in vivo* cell tracking tasks due to their excellent biocompatibility and comparable fluorescent stability 92-94. However, most organic luminogens face the challenge of aggregation caused quenching (ACQ) due to π-π stacking of hydrophobic organic dyes in aqueous media 95, which is a main obstacle in construction of high-performance fluorescent nanoparticles. Opposing to the ACQ effect, luminogens with aggregation-induced emission (AIE) characteristics exhibit bright fluorescence in aggregate state and weak fluorescence in molecular state 25,96-99. With the discovery of AIE effect, organic luminogens have been held up to a new level in biomedical application, especially in cell imaging and tracking 21,100-104. Recently, Tang *et al.* synthesized an AIEgen (BPN-BBTD) and prepared its NPs through encapsulation in amphiphilic Pluronic F-127 105. The BPN-BBTD NPs showed a broad emission spectrum covering 800 to 1300 nm with a QY (~1.8%) in the second near-infrared (NIR-II) window (>1000 nm), and a photothermal conversion efficiency of 39.8% under a 785 nm laser irradiation (**Figure 6**). Upon tail vein injection of NPs, the capillary with a small diameter of ~0.37 mm can be detected through NIR-II fluorescence imaging with a high spatial resolution. The NPs can preferably accumulate in tumor sites 24 h post intravenous injection in the subcutaneous and orthotopic bladder tumor-bearing mice, allowing effective photothermal ablation of tumors upon 785 nm laser irradiation (0.6 W cm^-2^). Noteworthy is that the intense and stable NIR-II fluorescence of BPN-BBTD NPs can ensure long-term tracking of bladder tumors for 32 days without any abnormalities or lesions in the main organs.

**Figure 7:**
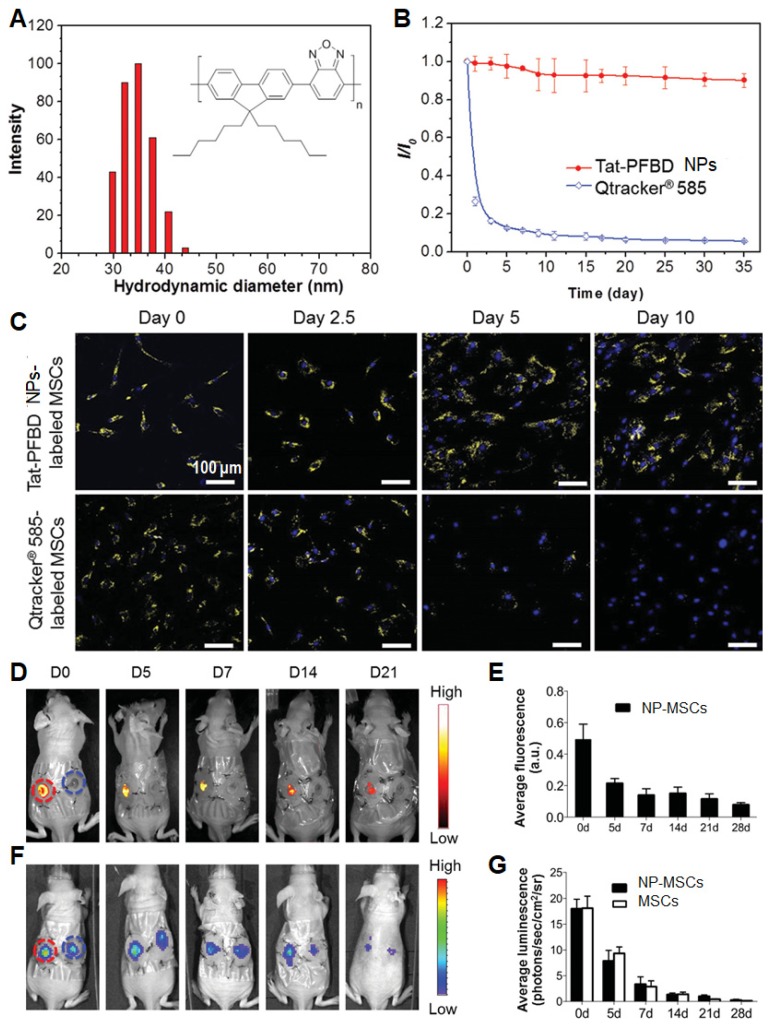
(A) Particle size distribution of Tat-PFBD NPs studied by DLS. The inset shows chemical structure of PFBD. (B) Time-dependent fluorescence intensity of Tat-PFBD (4 nM) and Qtracker^®^ 585 after culturing at 37 ℃. (C) The confocal images from MSCs labeled with Tat-PFBD and Qtracker^®^ 585 (*λ*_ex_: 488 nm; *λ*_em_: 550-780 nm). The nuclei were stained with DAPI (*λ*_ex_: 405 nm; *λ*_em_: 430-470 nm). (D) Representative *in vivo* fluorescence images of the wound sites on mouse transplanted with 1 × 10^6^ GFP/luciferase double-expressing MSCs with/without Tat-PFBD NP labeling (left- and right-side wound). (E) Time-dependent fluorescence intensity of the region of interests as marked in (D). (F) Corresponding *in vivo* luminescence images of the same mouse in (D). (G) Time-dependent bioluminescence intensity of the region of interests as marked in (F). Reprinted with permission from 64, copyright 2015 WILEY-VCH.

In addition to AIEgens, semiconducting polymers (SPs) represent another category of materials for synthesis of advanced fluorescent NPs. Their good photostability and large absorption cross-section will greatly benefit long-term biological studies. In this contribution, Li *et al.* used SP-based NPs (Tat-PFBD) as noninvasive fluorescent trackers with high brightness and low cytotoxicity for *in vivo* cell tracking to reveal the mechanism of transplanted MSCs in promoting skin regeneration 64,106,107. The obtained Tat-PFBD NPs had an average size of 37 nm in water, showing an emission maximum at 583 nm with a quantum yield of 42%. The arithmetic average number of emitted photons from each individual Tat-PFBD NP was determined to be 9.72 × 10^5^ counts per 100 s, which can promote the real-time single dot tracking and long-term bioimaging applications. After continuous incubation at 37 °C in Dulbecco's modified Eagle's medium, the fluorescence intensity of Tat-PFBD NPs remained above 90% after 35 days, whereas that of the most widely used commercial QD-based tracker (Qtracker^®^ 585) dropped to only 10% after 9 days. Moreover, Tat-PFBD showed significantly higher labeling efficiency and better long-term tracking ability without compromising the features of MSCs in terms of proliferation, migration, differentiation, and secretum. As a result, Tat-PFBD NPs showed an excellent *in vivo* tracking for 21 days (**Figure 7**), revealing that the transplanted MSCs can promote skin regeneration mainly through paracrine signaling effect. Besides, they also realized far-red-absorbing and NIR-emissive PIDT-DBT-Tat NPs to monitor orthotopic liver tumor growth for more than 27 days in a real-time manner 108. Through both* in vitro* and *in vivo* results, PIDT-DBT-Tat NPs as fluorescent probes showed better performance as compared to commercial QD tracker (Qtracker^®^ 705), whose fluorescent signal disappeared at day 6 in the orthotopic liver tumor growth study.

### Fluorescent nanodiamonds (FNDs)

FNDs are unique because they contain a high-density ensemble of negatively charged nitrogen-vacancy (NV^-^) centers as built-in fluorophores, in which the maximum wavelengths of absorption and emission are at 550 and 700 nm, respectively 55. Based on the special NV^-^ centers, FNDs possess various inherent properties: (i) higher spatial resolution than normal fluorescent dyes, which is particularly suitable to monitor cellular and subcellular components 55; (ii) optically detected magnetic resonance (ODMR) character, which is contributed by a *sp*^3^ nano-carbon allotrope 55; (iii) three-dimensional (3D) lattice structure, which can benefit the coating of functional groups on FND surface 109; (iv) the size between 35 and 100 nm, which is suitable for cell imaging 55. Thanks to the perfect photostability without photobleaching in NV^-^ center and low background in their emission region, FNDs are ideal materials in long-term cell tracking with good biocompatibility to maintain the self-renewal and differentiation of stem cells 110.

**Figure 8:**
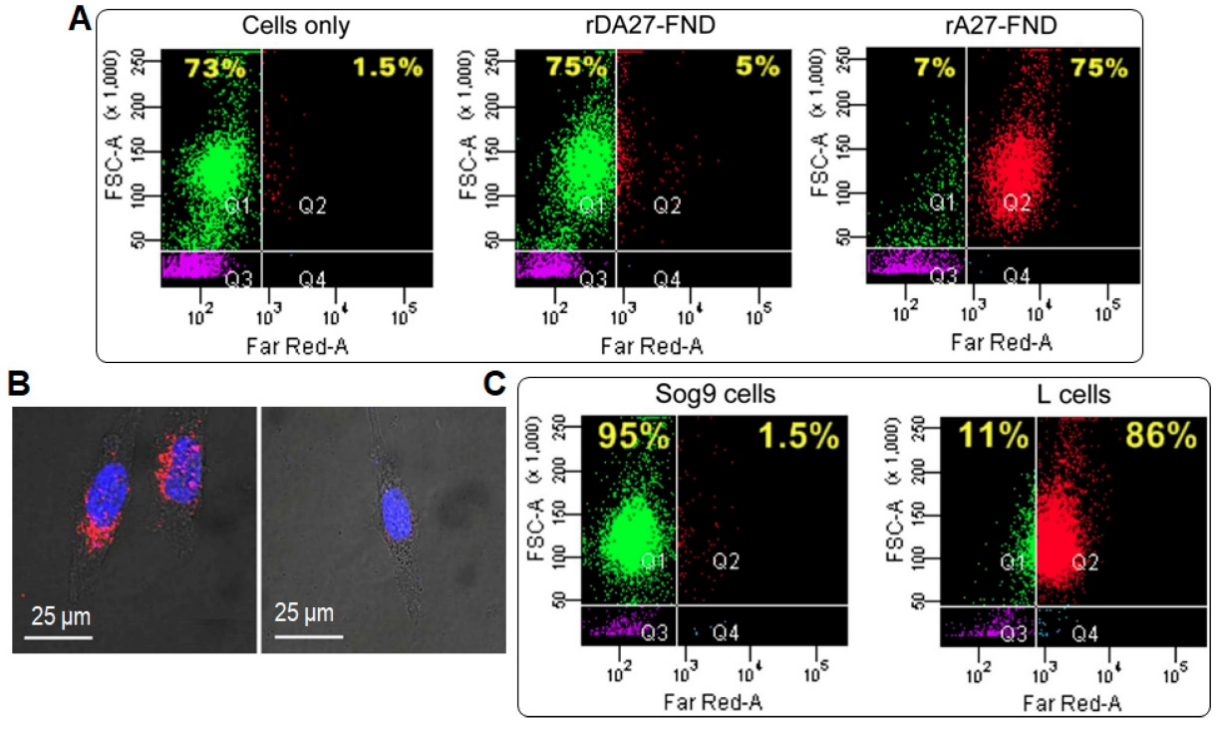
(A) Flow cytometric analysis of the cellular uptake of FNDs conjugated with rA27 and rDA27 (10 μg mL^‒1^) in comparison with HeLa cells only. (B) CLSM analysis of the cellular uptake of rA27-FNDs and rDA27-FNDs in HeLa cells. Red and blue colors stand for FNDs and Hoechst 33342 (nuclei tracker), respectively. (C) Flow cytometric analysis of the cellular uptake of rA27-FNDs (10 μg mL^‒1^) by mouse Sog9 and L cells expressing different levels of surface GAGs. Reprinted with permission from 112, copyright 2017 American Chemical Society.

Recently, Wang *et al.* targeted FNDs with transforming growth factor (TGF) to yield imaging probes for endogenous TGF-beta (TGF-*β*) receptor labeling and 3D single molecule imaging 111. To minimize aggregation, they used bovine serum albumin (BSA) to coat the FND surface *via* physical adsorption to reduce nonspecific interactions, yielding FND-TGF-BSA. The FND-TGF-BSA had an average hydrodynamic diameter of 46 nm in water, exhibiting strongly far-red fluorescence as well as high signal-to-noise ratio (SNR; ~48) passing through a single-band bandpass filter (675/67 nm). Interestingly, the probe can efficiently bind to HCC827 cells as a result of specific interaction with TGF-*β* receptor, demonstrating that FND-TGF-BSA is stable in live cells even under small molecule kinase inhibitor (SMI) treatment 111. As a result, the FNDs can be used as specific endogenous protein tags to study transmembrane signaling function and dynamics in 3D, which is useful in investigating the influence of drug on dynamic behaviors of the target proteins in living cells. Soon after, Chang *et al.* conjugated FNDs with a recombinant envelope protein of vaccinia virus (VacV) which has been used to track and image the glycosaminoglycans (GAGs) in targeted living cells (**Figure 8**) 112. Specifically, the recombinant A27 (rA27) proteins from VacV were used to functionalize FND surface because wild-type rA27 proteins (containing aa 21-84; named rA27-FND) are stable and can be easily produced in a large quantity 113. A mutant rA27 protein (containing aa 33-84) was employed to afford rDA27-FND as the negative control, due to its defective binding ability to heparan sulfates 114. Flow cytometric analysis revealed that about 75% of rA27-FND fed cells showed far-red emission owing to the specific targeting of GAG receptors by rA27-FND, whereas only 5% of rDA27-FND fed cells showed positive signals (**Figure 8A**). The rA27-FNDs thus showed more than 30-fold higher fluorescence intensity at far-red channel in living Hela cells as compared to that of rDA27-FNDs (**Figure 8B**). Similar results were observed when comparing the cellular internalization by GAG-overexpressed L cells (86% positive) and GAG-lacking Sog9 cells (1.5% positive) (**Figure 8C**). Furthermore, Schirhagl *et al.* took the advantage of unique optical property of FNDs to reveal the causes of cell aging at a molecular level and the exact function of free radicals in the aging process 115. Transmission electron microscopy (TEM) images revealed that FNDs were accumulated in cytoplasm around nucleus. The chronological life span assay suggested that FND internalization was effective to monitor free radicals in stationary phase yeast cell aging process, according to free radical theory of cellular aging 116.

**Figure 9:**
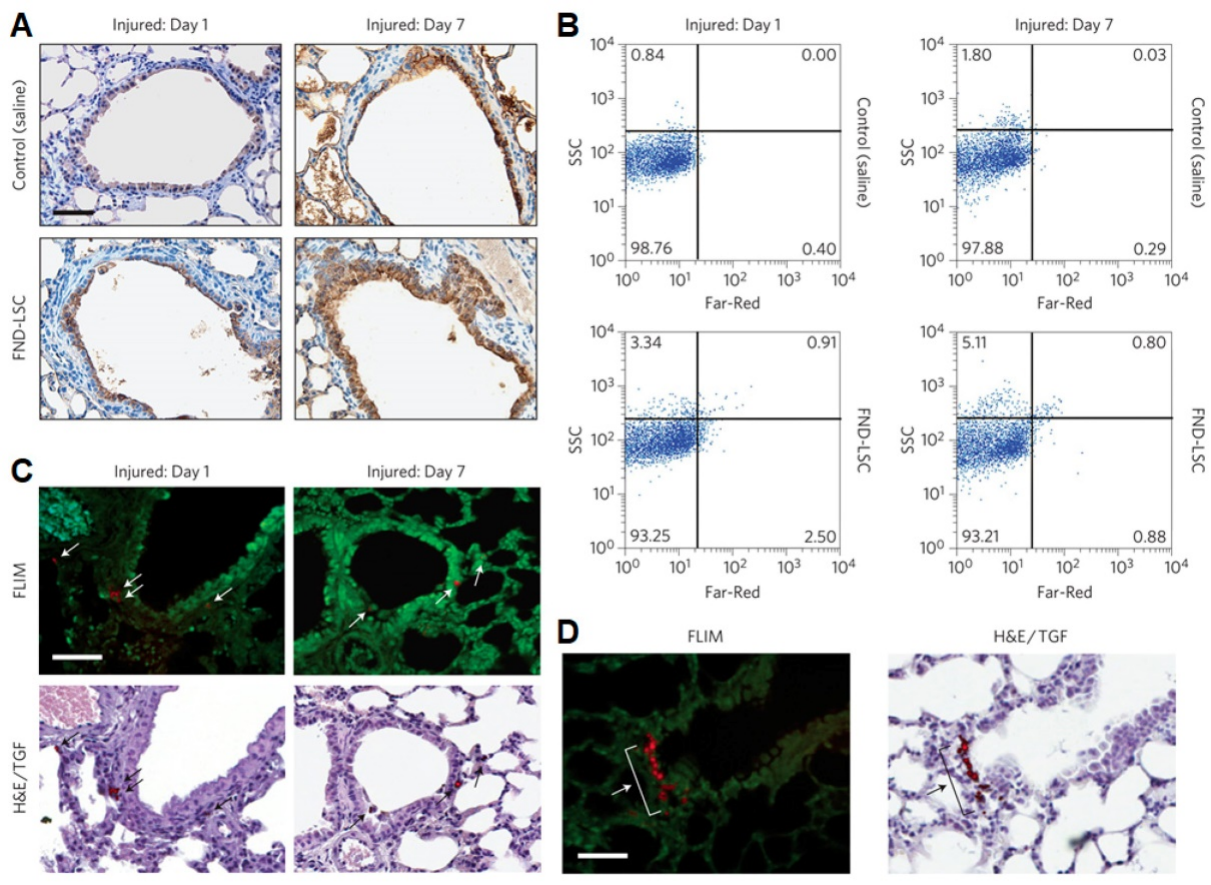
FND-labeled LSCs in lung-injured mice. (A, B) Immunohistochemical analysis of lung tissue sections (A) and flow cytometric analysis of total lung cells (B) collected from naphthalene-injured mice receiving an intravenous injection of saline (control) or FND-labeled LSCs for 1 and 7 days. The tissue sections in (A) were stained with CCSP for club cells (brown). (C) Representative FLIM, TGF and bright-field H&E staining images of the same lung tissue sections, showing the location of FND-labeled LSCs (white and black arrows) in terminal bronchioles of the lungs. (D) FLIM and H&E/TGF images of the lung tissue section from a naphthalene-injured mouse on day 7, showing engraftment of the transplanted FND-labeled LSCs (white and black arrows) to terminal bronchioles in a cluster formation. Scale bar is 50 μm. Reprinted with permission from 117, copyright 2013 Nature Publishing Group.

The excellent biocompatibility of FNDs is ideal for long-term cell tracking applications, particularly in stem cell research. Yu and Chang *et al.* used FND-labeled lung stem cells (LSCs) to identify their *in vivo* behaviors post transplantation and track the cellular engraftment and regenerative capabilities by fluorescence lifetime imaging microscopy (FLIM) (**Figure 9**) 117. Confocal fluorescence microscopy images revealed that LSCs labeled with FNDs did not show obvious difference from unlabeled LSCs in terms of self-renewal and differentiation ability. The result in time-gated fluorescence imaging of tissue section indicated that FDN-labeled LSCs trended to engraft at the terminal bronchioles of injury lungs after intravenous transplantation for 7 days. In addition, Chao *et al.* introduced FND-labeled embryonal carcinoma stem cells (ECSCs) to track their neuronal differentiation 118. After internalized by ECSCs, FNDs showed negligible effect on their proliferation and expression. Particularly, FNDs did not alter neuronal differentiation and neuron cells derived from ECSCs. In this contribution, the highly biocompatible FNDs could track neuronal differentiation processes for ~7 days using confocal microscope or flow cytometer.

**Figure 10:**
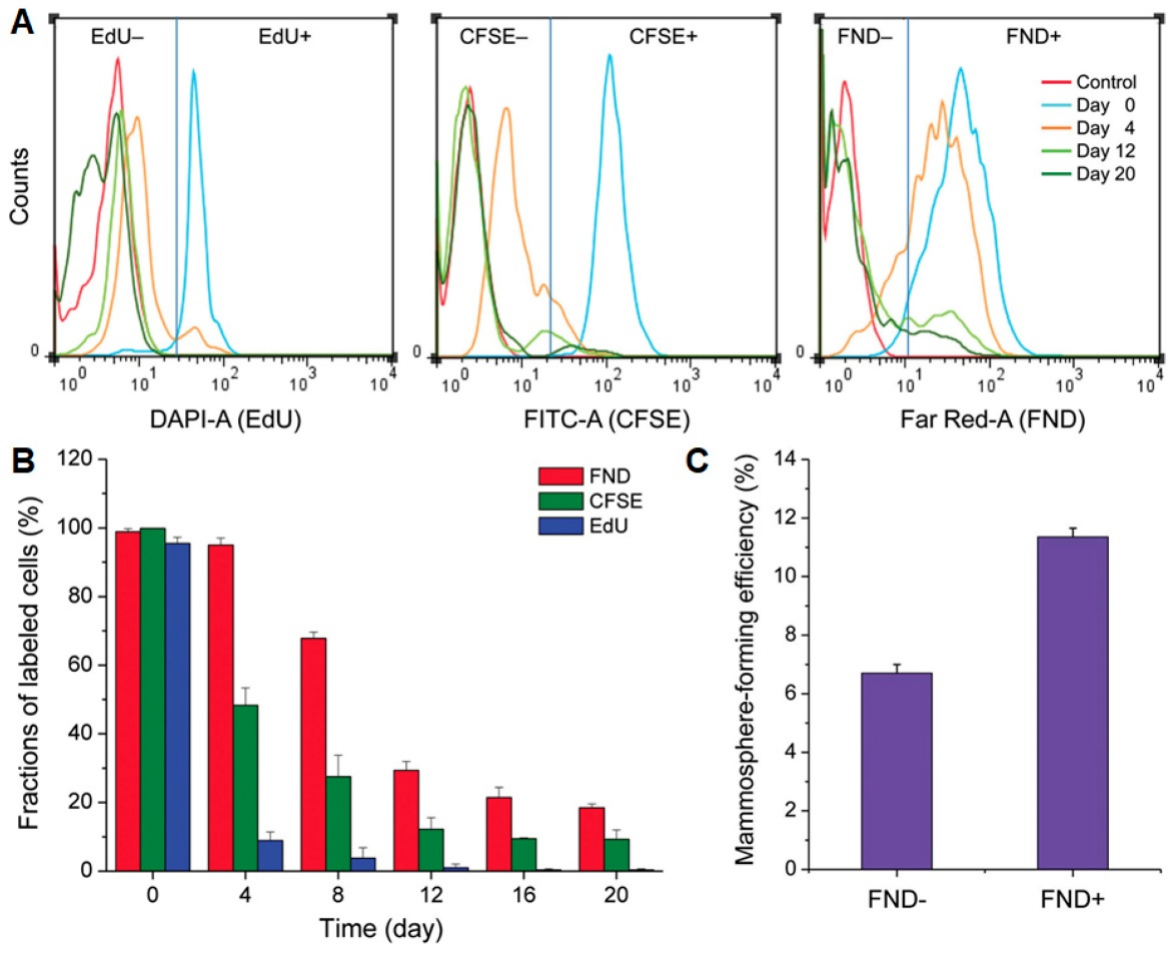
(A) Time-dependent flow cytometric analysis of dissociated mammospheres of AS-B145-1R cells after labeling with EdU, CFSE, and FND. (B) Comparison of the long-term tracking capability of EdU, CFSE, and FND, identified according to the flow cytometric analysis in (A). (C) Mammosphere-forming efficiencies of FND+ and FND- cells isolated from dissociated mammospheres formed by FND-labeled AS-B145-1R cells on day 7. Reprinted with permission from 119, copyright 2015 Wiley-VCH.

FNDs have also been used to track cancer stem cells (CSCs), which is considered as a source of tumor initiation 119. Geno-toxicity tests with comet and micronucleus assays for human fibroblasts and breast cancer cells indicated that FNDs had no influence on DNA or cell growth. After tracking human CSCs for 20 days, FNDs showed excellent intracellular retention capability, comparing with the commonly used cell trackers, carboxyfluorescein diacetate succinimidyl ester (CFSE) and 5-ethynyl-2′-deoxyuridine (EdU) (**Figure 10**). On day 4, flow cytometric analysis of dissociated mammospheres of AS-B145-1R cells showed a proportion of 9.5%, 12.4%, and 82.5% for EdU+, CFSE+, and FND+ cells, respectively. Specifically, 5.0% CFSE+ cells and 10.6 % FND+ cells were still detectable on day 20, whereas no EdU+ cells could be found by day 12 (**Figure 10B**). Interestingly, FND+ cells showed a mammosphere-forming efficiency nearly twice as high as that of the FND- cells (**Figure 10C**), resulting from slow-proliferating and/or quiescent CSCs for the former. These results indicated that FND-based cell tracking platform is an effective tool in distinguishing fast-proliferating and slow-proliferating/quiescent cells as well as tracking the clonal expansion of CSCs or other stem cells.

### Cell tracking *via* nuclear imaging

A combination technology of positive emission tomography (PET) and single-photon emission computed tomography (SPECT) for cell tracking is used to record functional processes in cells by detecting biologically active radiotracers, which can provide an image of targeted distribution 120. PET/SPECT lacks spatial resolution and cannot provide anatomic information, and they thus are usually combined with computed tomography (CT) technique 121 to precisely monitor the location of tracked cells 120. The cell tracking through PET/SPECT can be realized through either imaging intracellular reporter genes with radioactive reporter probes or labeling of cells with radioactive isotope-incorporated molecules or NPs 122.

**Figure 11:**
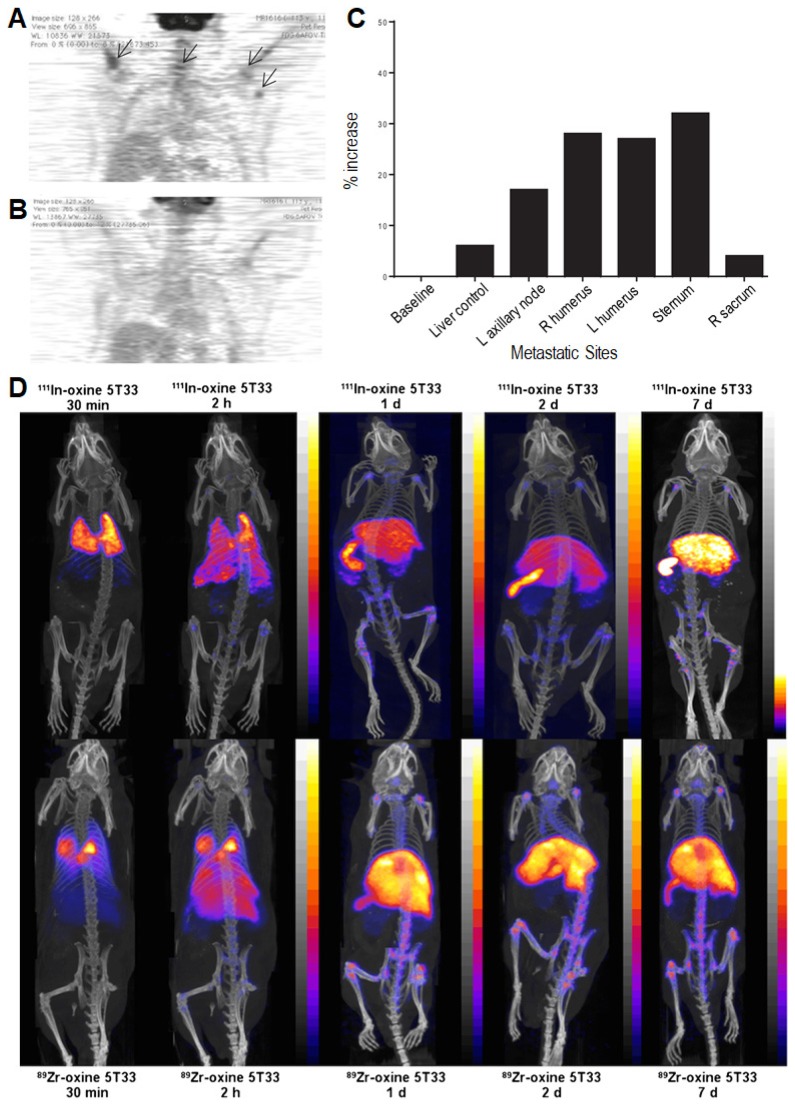
FDG PET/CT images of mice 48 h (A) and 3 months (B) post T-cell infusion. (C) FDG uptake over a period of 48 h post T-cell infusion. Reprinted with permission from 124, copyright 2016 BioMed Central Ltd. (D) PET/CT and SPECT/CT images of C57Bl/KaLwRij mice inoculated with [^89^Zr]oxinate_4_- or [^111^In]oxinate_3_-labeled eGFP-5T33 cells. Reprinted with permission from 127, copyright Springer Verlag.

Although T-cell therapy has made a wide impact on clinic, monitoring the fate of T cells remains a major challenge in cancer immunotherapy research. Thus, tracking transplantation of T-cells *in vivo* is necessary to evaluate the ability of T-cells to penetrate and traffic to tumors. Recently, Wu *et al.* labeled ^89^Zr-desferrioxamine with anti-CD8 cys-diabody (named ^89^Zr-malDFO-169 cDb) to track endogenous CD8^+^ T cells *via* noninvasive immune-PET 123. The 169 cDb can bind to CD8α, which is expressed on cytotoxic lymphocytes of all mouse strains, to realize lymph node and spleen targeting. To overcome the limitation of small region detection using PET, anti-CD8 immuno-PET based on ^89^Zr-malDFO-169 cDb not only detected lymph nodes in mice *via* targeting tumor-infiltrating CD8^+^ T cells, but also provided a preclinical evaluation for antitumor immune responses of immunotherapy through tracking dynamic distribution of T-cells. Disis *et al.* used ^111^In labeled HER-2/neu specific T-cells to monitor egress and traffic of the specific T-cells as assessed by concurrent SPECT/PET-CT imaging 124. The results showed a fluorodeoxyglucose flare at metastatic site and an increase of tumor uptake to 32% at 48 h post T-cell infusion (**Figure 11A-C**). This result provided evidence of T-cell homing to disease sites and a tumor metabolism flare response. Moreover, Weber *et al.* utilized ^86^Y/^177^Lu-AABD to continuously track DAbR1-positive chimeric antigen receptor T (CAR T) cells *via* PET/SPECT 125. They observed that ^86^Y-AABD could be maintained in T cells and cleared by normal tissue, which ensured a high contrast of* in vivo* cell tracking *via* PET and SPECT.

In addition, Blower *et al.* designed a [^89^Zr]oxinate_4_ PET tracer to monitor eGFP-5T33 murine myeloma cells 126 for 14 days 127. Compared with [^111^In]oxinate_3_, [^89^Zr]oxinate_4_ had a better intracellular retention ability (>90%) after 24 hours. Using eGFP-positive cells, the results showed that the translocation of radioactivity to kidneys was much greater for [^111^In]oxinate_3_, while more than 92% of [^89^Zr]oxinate_4_ remained associated with the cells in liver, spleen and bone marrow after 7 days *in vivo* (**Figure 11D**). These results indicated that [^89^Zr]oxinate_4_ was a potential long-term PET tracer to observe survival and behavior of different cell types. Soon after, Fruhwirth *et al.* verified a novel PET radiotracer, [^18^F]tetrafluoroborate ([^18^F]BF_4_^‒^), as a reporter to detect orthotopic xenograft breast cancer model expressing the human sodium iodide symporter (NIS) 128. For NIS-afforded *in vivo* tumor metastasis detection, [^18^F]BF_4_^‒^-PET images showed significant radiotracer uptake in the primary tumors and axillary and inguinal lymph nodes. Compared with traditional [^123^I] radiotracer, [^18^F]BF_4_^‒^ was highly specific and sensitive to track cancer cells with better pharmacokinetics (*e.g.*, faster tumor uptake, quicker and more complete clearance from circulation). Accordingly, [^18^F]BF_4_^‒^ *via* PET/CT imaging has a preclinical potential in* in vivo* cell tracking for NIS expressing disease models.

### Cell tracking *via* photoacoustic imaging

Photoacoustic (PA) imaging as a noninvasive imaging modality has great potential in biomedical and clinical applications, owing to its unique advantages of superb contrast, high spatial resolution, and high sensitivity to tissue functional characteristics 129-131. PA signals are similar to ultrasound (US) waves in imaging, where the light scattering and dissipation by tissue can be minimized. PA imaging thus can afford deeper tissue penetration in comparison with other optical imaging technologies. However, to increase the light intensity and PA SNR in deep tissues, PA-imaging contrast agents (PA-trackers) have only been developed in recent years 132. An excellent PA-trackers should own special photophysical properties, such as low fluorescence quantum yield, high molar-extinction coefficient in the near-infrared (NIR) window absorption, and good photostability. To date, a variety of PA-trackers have been developed, including carbon nanotubes, graphene-based agents, gold nanorods, organic small molecules, and SPNs.

**Figure 12:**
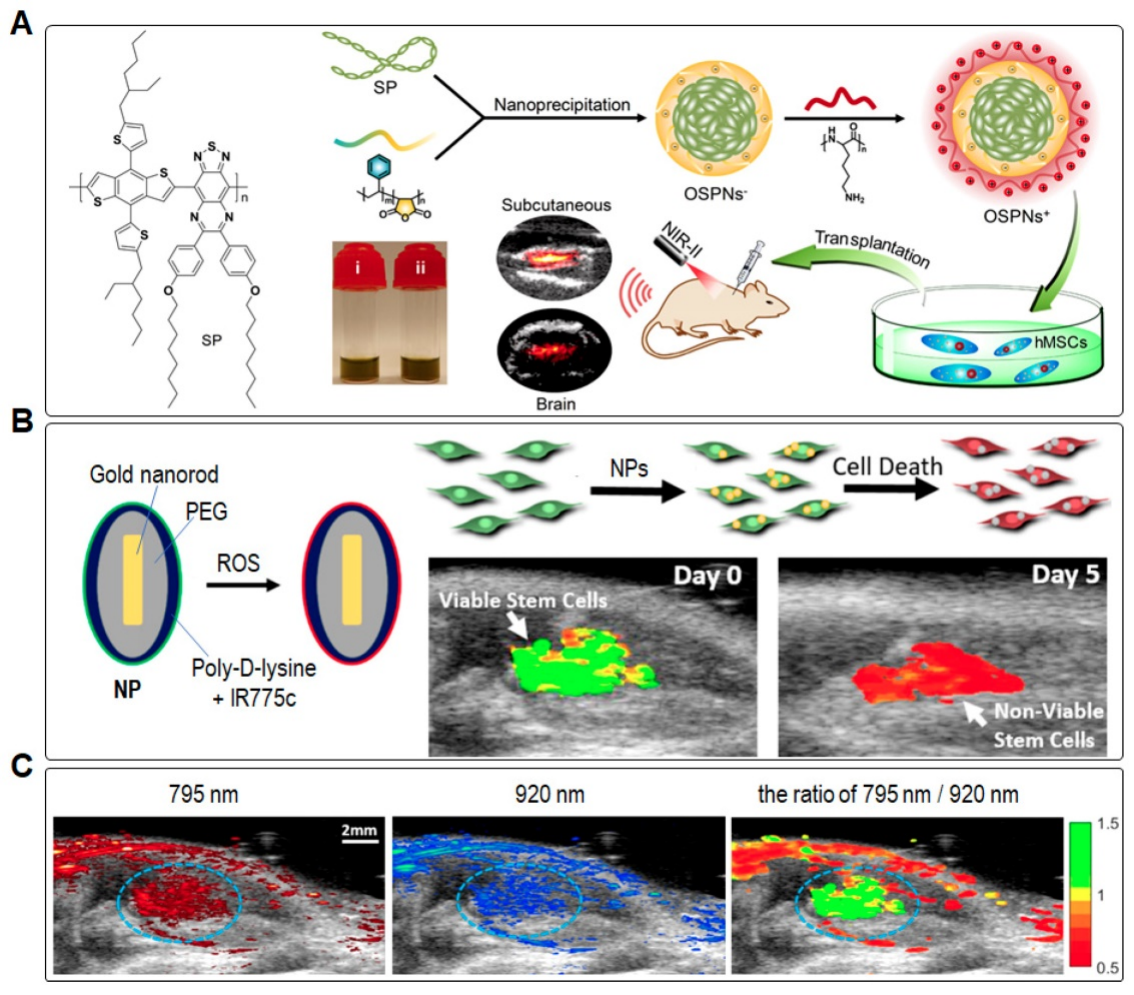
(A) Schematic illustration of the preparation procedure of OSPNs^+^ and PA labeling of hMSCs for transplantation. Reprinted with permission from 14, copyright 2018 American Chemical Society. (B) *In vivo* PA tracking of cell viability based on a ROS-sensitive NP, and (C) *in vivo* US/PA imaging of transplanted stem cells on day 0, correlating with the IR775c (*λ*_ex_: 795 nm) and AuNRs (*λ*_ex_: 920 nm) emission intensities. Reprinted with permission from 17, copyright 2019 American Chemical Society.

For the recent cell-tracking using PA imaging, Wu and Li *et al.* developed a NIR-absorbing SP-based PA tracker to monitor the transplantation of human embryonic stem cell-derived cardiomy-ocytes (hESC-CMs) in living mouse hearts 18. The PA tracker exhibited an excellent sensitivity in detecting 2000 labeled CMs to distinguish them from background tissues without significant impact on the structure and function of hESC-CMs. Moreover, the high labeling efficiency and PA signal allowed assess the delivery and engraftment of hESC-CMs in beating hearts, providing a facile approach to evaluate the efficacy of cell transplantation in cardiac regenerative therapy. Later, Bian *et al.* used a second near-infrared (NIR-II) absorptive organic SP-based nanoprobe (OSPN) as PA tracker to label hMSCs for *in vivo* tracking (**Figure 12A**) 14. PA signal from the OSPNs in cytoplasm was measured by optical-resolution photoacoustic microscopy (OR-PAM), revealing that hMSCs had more efficient uptake of positively charged OSPNs^+^ than that of the negatively charged OSPNs^-^. Quantitative analysis revealed that the PA signal intensity of OSPNs^+^-labeled cells was 3-fold higher than that of OSPNs^-^-labeled cells. In addition, PA imaging excited by NIR-II light irradiation (1064 nm) showed higher SNR (1.4-fold) in comparison with that excited by the first near-infrared (NIR-I) light irradiation (860 nm) at the same power intensity (5.0 mJ cm^-2^), implying that OSPN^+^ was superior in deep brain imaging with NIR-II absorption. Noteworthy is that the PA contrast was still visualized clearly upon 2 weeks post-injection, even the intensity decreased by ~61.4% as a result of cell proliferation. Thus, the NIR-II SPN-based PA-trackers were demonstrated to show significant advantages for labeling and tracking of hMSCs in biological tissue, providing a powerful method to understand the mechanisms in stem cell therapy.

In addition, Suggs* et al.* used inert gold nanorods (AuNRs) coated with a reactive oxygen species (ROS) sensitive NIR dye (IR775c) as the PA-tracker to label and track the viability of MSCs (**Figure 12B**) 17. After cell death, MSCs produced ROS to degrade the cells. Accordingly, the viability of MSCs can be measured by PA signal ratio of IR775c (795 nm) to AuNRs (920 nm) because the latter are insensitive to ROS. The ratiometric heatmap of PA signal revealed the location of MSCs, where the high ratio value indicated living MSC populations while the low ratio value implied dying or dead cell populations (**Figure 12C**). After transplanted into the gastrocnemius muscle, the PA intensity of MSCs for IR775c decreased by ~22% at day 1 and ~38% at day 10 based on the normalized intensity for AuNRs. As a result, the relative viability of MSCs was estimated to be 0.92 and 0.05 for day 0 and day 10, respectively. In addition, PA imaging results revealed that the viability of cell population within the first day post transplantation decreased rapidly. Such ROS-sensitive PA-tracker can provide a faster process to optimize their therapeutic condition in a preclinical research, including cell transplantation timing, number of transplanted stem cells, and stem cell viability after administration.

**Figure 13:**
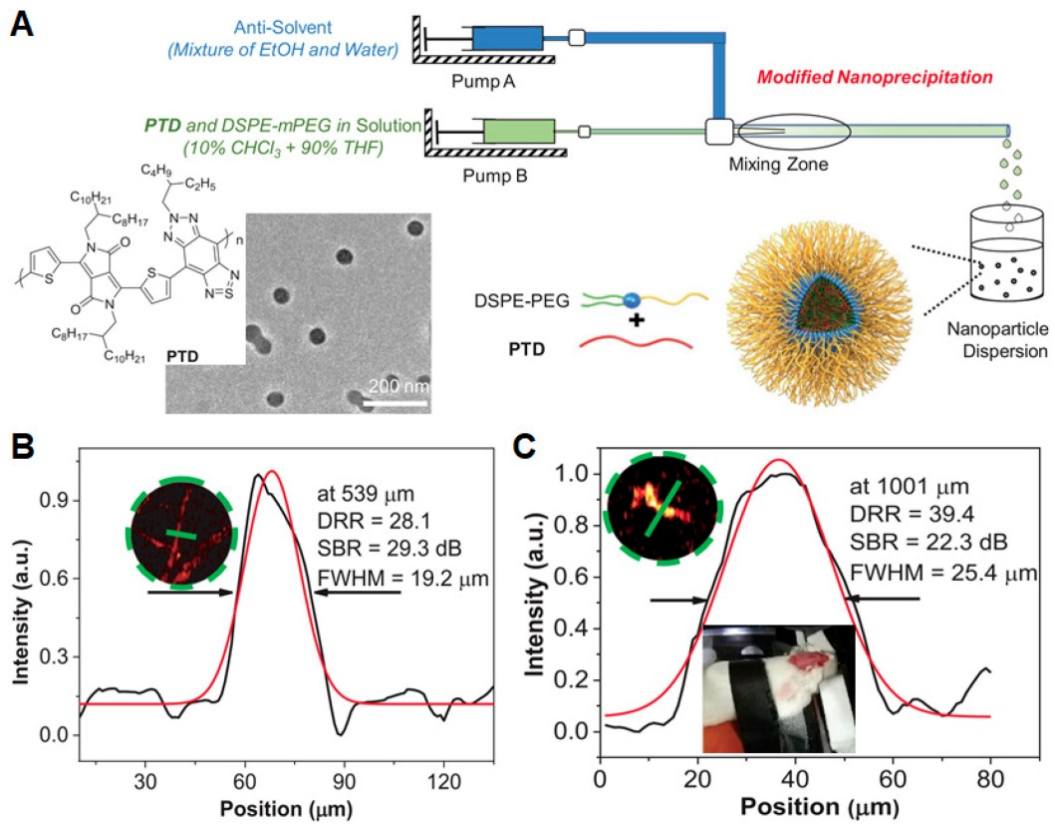
(A) Schematic diagram of microfluidic glass capillary mixer for the synthesis of monodisperse PTD NPs through modified nanoprecipitation. (B and C) PA intensity profiles for the inset zoomed areas of mouse ear (B) and brain (C) vasculature images at the depths of 539 and 1001 µm. Reprinted with permission from 133, copyright 2019 WILEY-VCH.

Blood vessels play a crucial role in living bodies as a physical barrier and functional network, which facilitate the transportation of solutes, nutrients, and cells among tissues. A number of diseases involve the disfunction of blood vessel. As such, tracking vascular structures by imaging is of high important in theranostic applications. Recently, Liu *et al.* developed a NIR-II absorptive conjugated polymer (PTD) and fabricated PTD NPs by a modified nanoprecipitation method using a coaxial microfluidic glass capillary mixer (**Figure 13A**) 133. Under optimized microfluidic conditions (320 of Reynolds number and 40% EtOH), the formulated NPs exhibited the smallest average diameter of 40 nm by TEM image. PTD NPs showed a strong NIR-II absorption peak at 1160 nm with an extinction coefficient of 48.1 L g^-1^ cm^-1^ in aqueous media, which could contribute to their strong PA amplitude under a pulsed laser irradiation of 1064 nm. To evaluate *in vivo* angiography performance of PTD NPs, 3D OR-PAM imaging, a decipherable technology for wide-field 3D biological structures with deep penetration and large signal-to-background ratio (SBR), was constructed on a mice ear and cerebral vasculatures with an excitation wavelength of 1064 nm. The regular ear vasculatures with a resolution of 19.2 µm and an SBR of 29.3 dB at the maximal imaging depth of 539 µm were visualized clearly (**Figure 13B**). Moreover, 3D whole-cortex cerebral vasculatures with a large imaging area (48 mm^2^), good resolution (25.4 µm), and high SBR (22.3 dB) at a depth up to 1001 µm are also clearly resolved through the intact skull (**Figure 13C**). These results suggested that ORPAMI using NIR-II NPs as PA-trackers will be a competitive imaging technology in enhancing the spatiotemporal resolution of cell-tracking in future. In addition, Liu and Zheng *et al.* developed AIE dots to achieve precise brain cancer diagnostics. The AIE dots showed an NIR-I absorption maximum at 740 nm with a large absorptivity (10.2 L g^-1^ cm^-1^) and NIR-II emission maximum at 975 nm with a high quantum yield (6.2%), promoting the synergetic NIR-I photoacoustic and NIR-II fluorescence imaging 134. Among them, NIR-II fluorescence imaging showed a high SBR (4.4) and resolution (38 µm), whereas PA signal of tumor region could reach 2.0 mm of tissue depth. Collectively, the synergetic PA and fluorescence imaging based on AIE dots in NIR window will own great potential for monitoring and visualizing the abnormalities in blood vessel and tissue of brain toward precise brain-tumor diagnosis 135.

## Theranostic applications of multifunctional trackers

In addition to cell tracking, NPs can also serve as theranostic agents for disease diagnosis and treatments. For example, some luminogens can generate ROS or heat upon light irradiation, which have the capability to ablate cancer cells, simultaneously realizing both diagnostic and therapeutic effects 136,137. Besides, many types of leukocytes, including macrophages, neutrophils, and dendritic cells, can sense chemokine and cytokine cues and thus can play the role as nanocarriers to target tumors 138,139. Inspired by this concept, Xie and Li *et al.* developed silica nanocapsules, consisting of doxorubicin (Dox) as a representative drug, IONs as MRI tracker, ^63^Cu-PTSM as PET tracker and DiD dye as fluorescent tracker (named DSN). The DSNs can be efficiently loaded into macrophages. After intravenous injection, the DSN-laden macrophages showed high accumulation in tumor and further efficiently suppressed tumor growth upon drug release in situ with low systematic toxicity 140. As such, the multifunctional cell trackers can be used to carry therapeutic agents and track the loaded cells simultaneously, opening a new door in disease theranostics. In this section, the new treatment methods based on NPs will be discussed, mainly covering photodynamic therapy (PDT) 141 and photothermal therapy (PTT) 62,142. Inspired by the pioneering studies, further combination of cell trackers with novel therapeutic modalities will motivate new research activities in biomedical studies.

### Photodynamic therapy (PDT)

**Figure 14:**
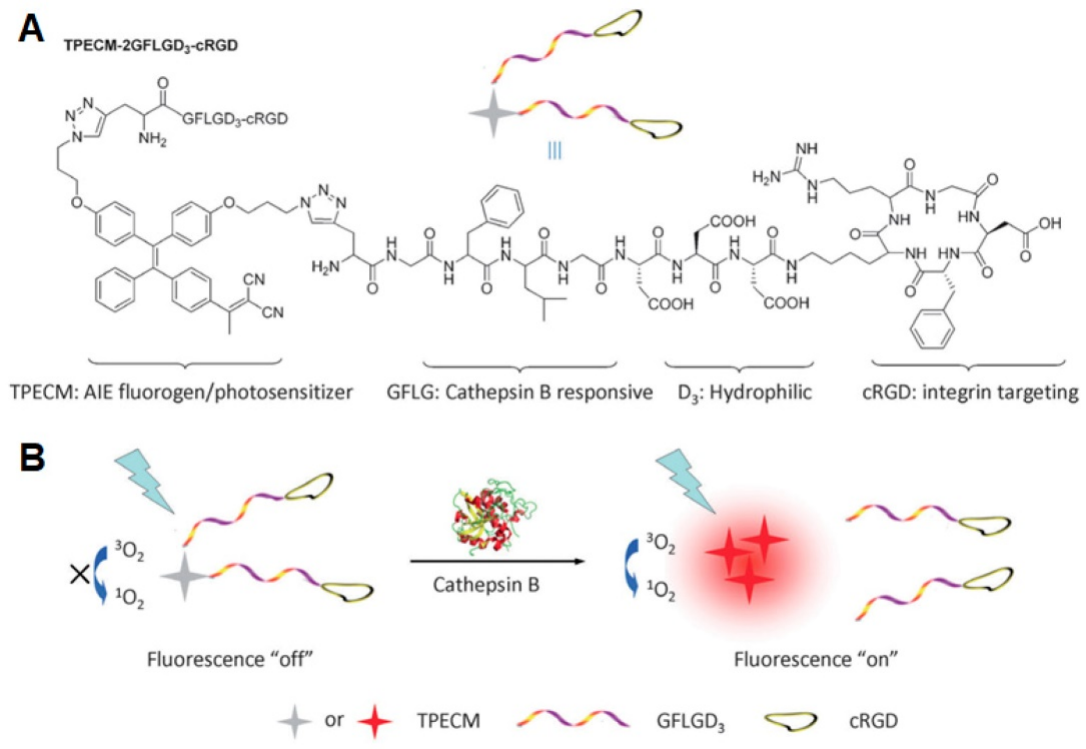
(A) Schematic illustration of TPECM-2GFLGD_3_-cRGD bioprobe. (B) Probe activation by cathepsin B with fluorescence “turn-on” and activated photoactivity to generate ROS upon light irradiation. Reprinted with permission from 151, copyright 2015 WILEY-VCH.

**Figure 15:**
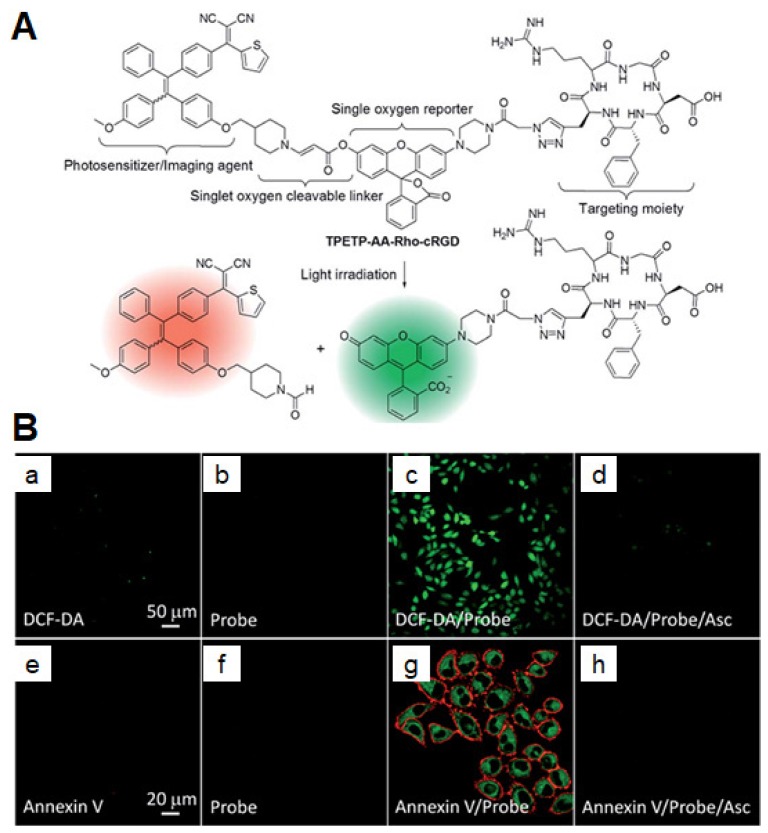
(A) The chemical structure of TPETP-AA-Rho-cRGD and its real-time PDT cell tracking illustration. (B) CLSM images showing ROS generation of TPETP in MDA-MB-231 cells after different treatments using DCF-DA indicator (2 μM; *λ*_ex_: 488 nm, *λ*_em_: 505-525 nm) (a-d) and annexin V-Cy5 indicator (1 μM; *λ*_ex_: 633 nm, *λ*_em_: >650 nm) (e-h) with and without a singlet oxygen scavenger ascorbic acid (Asc). Green fluorescence (Rho; *λ*_ex_: 488 nm, *λ*_em_: 505-525 nm). Reprinted with permission from 156, copyright 2016 Royal Society of Chemistry.

Photosensitizers (PSs) generating ROS have attracted a broad research interest in different application fields, including PDT 143, organic synthesis 144, and sewage wastewater treatment 145,146. PDT denotes a well-consolidated but gradually expanding approach in the cancer treatment, owing to its high spatiotemporal precision, controllability, and noninvasive nature 147. To enhance the accuracy of therapy, Liu *et al.* developed an activatable AIE PS for the simultaneous imaging and photodynamic ablation of cancer cells 148. Upon conjugation to cRGD 22,149 with an enzyme-activatable spacer 150,151, PSs simultaneously showed light-up fluorescence imaging and activated PDT for specific cancer cells (**Figure 14**) 152. In this work, the PSs could specifically target lysosomes of MDA-MB-231 cells overexpressing α_v_β_3_ integrin rather than MCF-7 and 293T cells. After PSs were activated by cathepsin B, a lysosomal protease, under light irradiation, the aggregation of released AIEgens showed a fluorescence turn-on signature. This process further facilitated the ROS production to damage the lysosomal membranes and cause cell apoptosis, showing considerable potentials for targeted and image-guided PDT 151,153-155. Later, the same group synthesized a self-reporting AIE nanoprobe for real-time monitoring ROS and self-tracking photodynamic ablation of cancer cells 156. The AIE molecule (named TPETP-AA-Rho-cRGD) composed of TPETP as an AIE luminogen (AIEgen), aminoacrylate (AA) as a singlet oxygen cleavable linker, a fluorogenic rhodol dye (Rho), and cRGD as targeting group (**Figure 15**). They self-assembled into NPs in a mixture of DMSO and phosphate buffered saline (1/199, v/v) with an average size of ~140 nm, exhibiting a high singlet oxygen QY of 0.68 157. Under light irradiation, the AA linker was cleaved as a result of ROS generation and green fluorescence from rhodol dye was then observed. Upon imaging-guided photodynamic therapy (PDT), the nanoprobe can efficiently monitor singlet oxygen generation *via* fluorescent signal changes.

**Figure 16:**
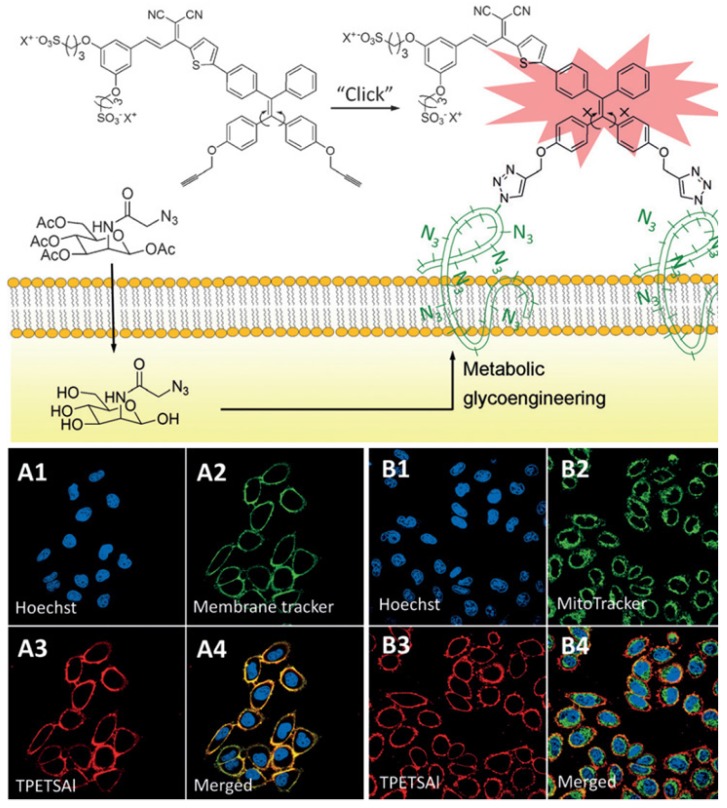
The bioorthogonal turn-on probe for cancer cell imaging. CLSM images of HeLa cells co-stained with TPETSAl and membrane tracker CellMask Green (A1-A4) or MitoTracker Green (B1-B4). Blue fluorescence (nuclei live dyed with Hoechst 33342, *λ*_ex_: 405 nm, *λ*_em_: 430-470 nm); green fluorescence (MitoTracker Green or CellMask Green, *λ*_ex_: 488 nm, *λ*_em_: 505-525 nm); red fluorescence (TPETSAl, *λ*_ex_: 488 nm, *λ*_em_: >650 nm). Reprinted with permission from 164, copyright 2016 WILEY-VCH.

In clinical study, especially in fluorescence-guided surgery 158,159, the implementation of highly sensitive and specific imaging modalities for timely cancer diagnosis and progression monitoring has been of great significance. Unlike traditional methods of endogenous targeting molecules (antibodies, peptides, or aptamers) labeled with imaging probes 160,161, bioorthogonal chemistry has shown powerful applications in biological fields in combination with metabolic glycoengineering. Through the intrinsic metabolism of metabolic glycoengineering, the cancer cell surface labeling with unnatural sialic acids can be specifically targeted through chemically binding of *in vivo* biorthogonal click chemistry 162-170. Inspired by the unique properties of AIEgens, Liu *et al.* developed a water-soluble AIEgen tying triple-bond, a reactive site with azido-functionalized glycans on cancer cell surface, to achieve bioorthogonal turn-on imaging (**Figure 16**) 164. In aqueous media, the bioorthogonal probes showed very weak fluorescence. However, a “turn-on” red fluorescence was clearly observed upon click reaction with azido-functionalized cancer surface owing to the restriction of intramolecular motion. Moreover, photodynamic ablation for targeting cancer cells can be further accomplished at the same time. Compared to previously reported bioorthogonal probes 167-169, AIEgen-based probes can realize a unique alternative for real-time live cell labeling, imaging, and therapy. To improve *in vivo* bioorthogonal efficiency, the copper-free click reactive probe 166,170 and cancer-cell-specific metabolic precursor 165 would be subsequently developed for *in vivo* labeling. These studies suggest that the bioorthogonal probes targeting to tumor cell surface with covalent bond can serve as long-term cell trackers as well as fluorescent guidance for clinical surgery.

### Photothermal therapy (PTT)

**Figure 17:**
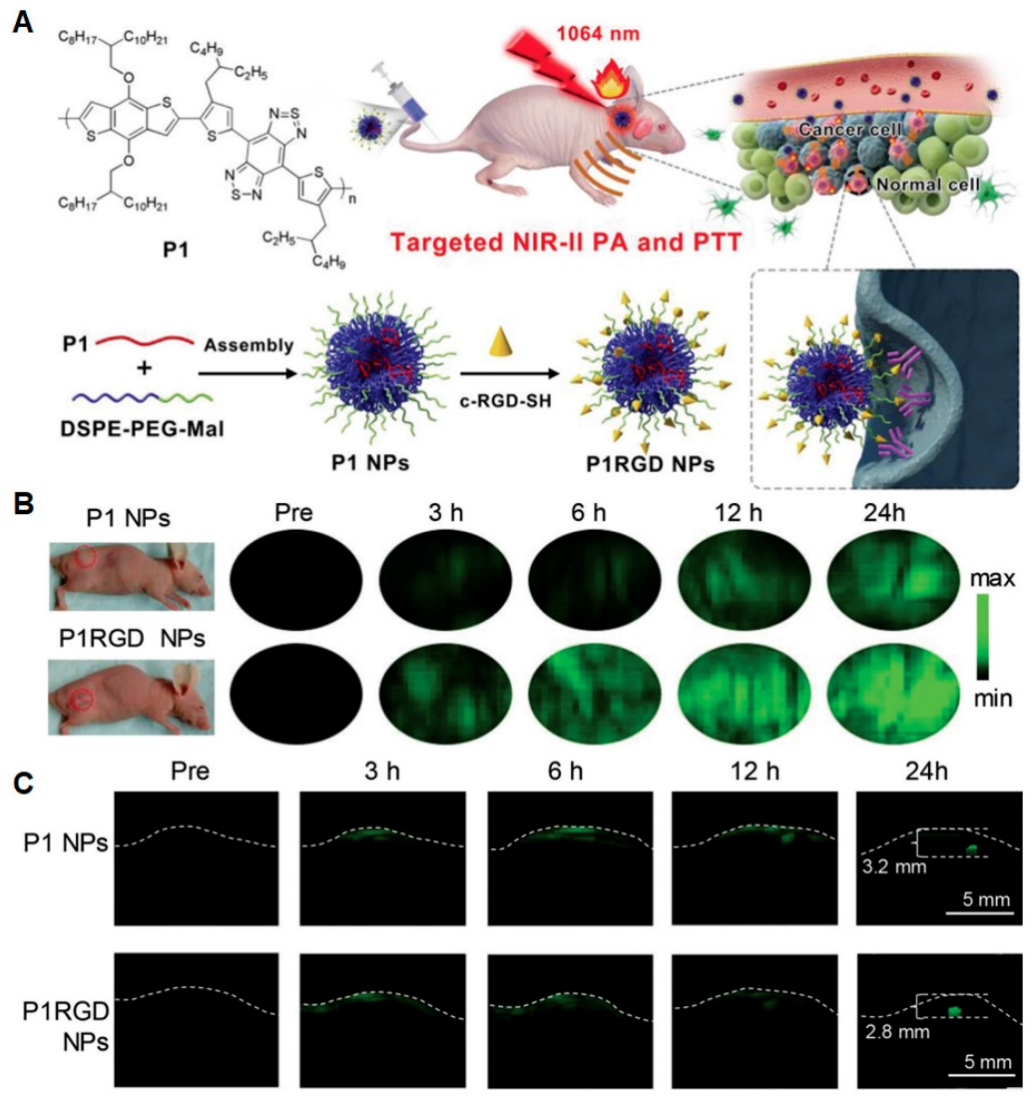
(A) Schematic illustration of NP synthesis and *in vivo* brain tumor photothermal therapy and PA imaging. (B) *In vivo* noninvasive PA imaging results of mice in subcutaneous U87 xenograft tumor model at different time points post NP injection. (C) Through scalp and skull noninvasive PA imaging of mouse brain tumors at different time points upon NP injection. Reprinted with permission from 181, copyright 2018 WILEY-VCH.

Photothermal therapy (PTT), another light-activatable therapy, collocating PA or NIR-II imaging has exhibited advantages over conventional theranostics such as fluorescence-guided chemotherapy 15,16,171-182. PA-trackers naturally have photothermal functions, which thus can be regarded as PTT agents. They can synergistically offer deep penetration, high spatial resolution, and effective therapy with minimal side-effects. So far, various NIR-II PTT agents have been prepared from inorganic (copper sulfide, plasma metal clusters, and carbon nanotubes) 183-185 and organic materials (small molecules and semiconducting polymers) 186-188. The organic agents usually have relatively excellent biodegradation and biocompatibility, which are desired in *in vivo* biomedical applications 60. Compared to small molecules, donor-acceptor (D-A) structured SPs have advantages in good photostability and large extinction coefficient, which can benefit PTT efficacy 62,180-182. Hence, Liu and Zheng *et al.* developed NIR-II absorptive SPNs (named P1 NPs, 50-64 nm) with an active targeting cRGD ligand (named P1RGD NPs) for selective targeting of integrin on tumor cell surface (**Figure 17A**) 181. Under laser irradiation (1064 nm, 1 W cm^-2^), the temperature of P1 NPs (0.1 mg mL^-1^) rapidly increased to 64.8 ℃ within 5 min and the photothermal conversion efficiency (PTCE) was calculated to be 30.1%. In addition, PA signals of P1RGD NPs exhibited 3.5-fold higher than that of P1 NPs at tumor sites (**Figure 17B**), suggesting that cRGD did significantly enhance tumor uptake. For orthotopic tumor model (**Figure 17C**), upon 24 h post-injection, both P1 NPs and P1RGD NPs showed high PA signals in tumors with a detectable depth up to 3.2 and 2.8 mm, respectively. Moreover, P1RGD NP-based PA imaging performed a higher SBR of 90 in comparison with P1 NP-based one (SBR of 61), implying that active brain-tumor targeting could be realized by cRGD-modified NPs for improved therapeutic efficacy. This work demonstrated that photostable and biocompatible NIR-II SPNs will be promising for precise tracking as well as further treatment of brain tumors.

**Figure 18:**
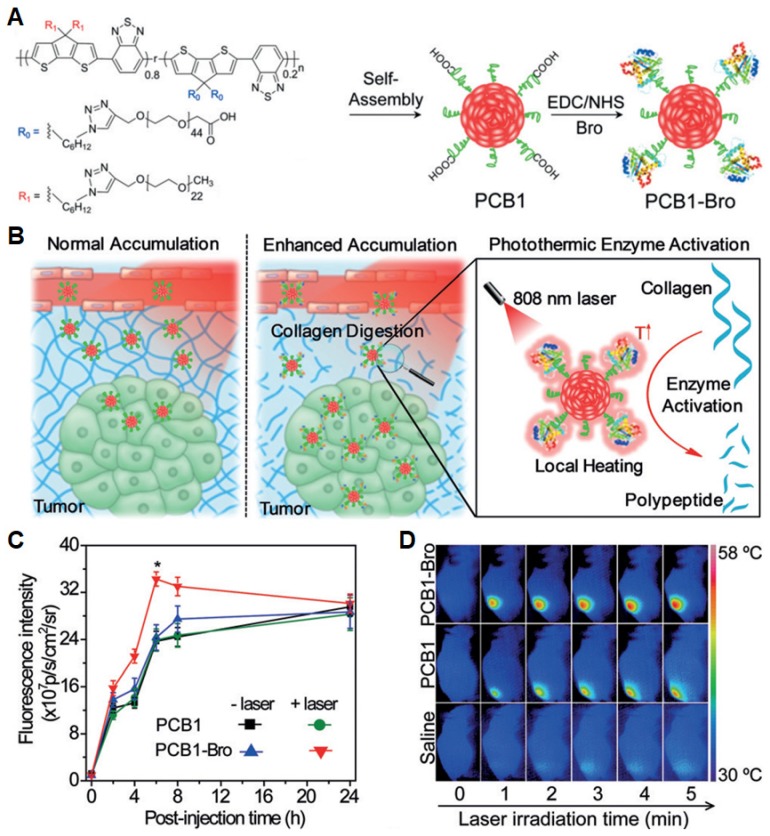
(A) Schematic illustration of PCB1-Bro NP and its synthetic strategy. (B) Schematic illustration of photothermally triggered enzyme activation of PCB1-Bro towards collagen digestion for enhanced accumulation of nanoparticles in tumors. (C) Fluorescence intensity of tumors as a function of post-injection time of PCB1 or PCB1-Bro with and without laser irradiation. (D) IR thermal images of 4T1 tumor-bearing mice under laser irradiation for 5 min after intravenous injection of saline, PCB1, and PCB1-Bro, respectively. Reprinted with permission from 175, copyright 2018 WILEY-VCH.

In addition, to enhance accumulation of NPs in tumor, Pu* et al.* synthesized a NIR photothermal active SP nanoenzyme (named PCB1-Bro) (**Figure 18A**), which contained a SP amphiphile (PCB-1) and bromelain (Bro), serving as a photothermal nano-transducer and a temperature-sensitive enzyme, respectively 175. Under NIR laser irradiation, PCB1-Bro underwent efficient photothermal conversion to increase the temperature around tumor and activated Bro enzyme to digest the most abundant tumor extracellular-matrix proteins, leading to enhanced NPs accumulation in tumor with boosted PTT effect (**Figure 18B**). At 6 h post-injection, the fluorescence intensity from PCB1-Bro enriched in tumor reached a maximum value, which was 1.4-fold higher than other groups (**Figure 18C**). This can be attributed to the improved EPR effect after digesting collagen by enzymatic activation of PCB1-Bro. Consequently, PCB1-Bro completely inhibited the tumor growth for 16 d due to higher PTT efficacy (**Figure 18D**). Thus, the strategy of using nanoenzyme will provide an innovative approach to enhance the drug delivery efficacy for PTT and other therapies such as PDT, chemotherapy, and gene therapy 155.

## Conclusions and perspectives

In this review, we summarized the recent advances in exploration of nanoparticles as long-term cell trackers for cell therapies and cancer theranostics. The advantages and limitations of currently available cell trackers for various diagnostic technologies, including MRI, MPI, PA, PET, SPECT and fluorescence imaging, have been discussed and summarized in **Table 2**. Successive *in vitro* and *in vivo* cell tracking over a long period of time can provide valuable evidence on cellular processes and biomedicinal therapies, especially on stem cell therapy 189,190 and immunotherapy. Although the traditional clinical techniques such as MRI and PET/SPECT have excellent advantages in the near-ideal and/or limitless *in vivo* depth detection and quantitative analysis, they face the challenge of high cost and relatively slow imaging speed except for radioisotope safety. By contrast, PA and fluorescence imaging techniques are emerging research fields, enjoying much lower cost for imaging reagents, faster imaging processes within seconds-to-minutes and more maneuverable instruments. These trackers based on organic small-molecules and/or semiconducting polymers have advantages in *in vivo* real-time and long-term cell tracking, owing to their good biocompatibility. However, further efforts should be made to enhance the *in vivo* tissue penetration depth without compromising the imaging resolution, which is of high importance for quantitative analysis. To date, some promising results have been shown with the revolutionary development of imaging techniques from software to hardware. For instance, thanks to the minimized tissue absorbance and interference in NIR-II window, the problem of detection depth can be alleviated. Previous studies in NIR-II window have shown a penetration depth of 11.6 cm with 18 dB of SNR in PA imaging 191 and 1.3 μm with 12 of T/NT ratio for fluorescence imaging 192, which demonstrated significant superiority than NIR-I trackers. On the other hand, multi-modal NPs hold great potentials in deep tissue imaging and precise disease diagnosis, particularly in the cerebrovascular and brain tissues. Through combination of different imaging modalities, such multi-modal NPs can round off the strong points of each imaging technique to acquire more comprehensive information than single-modal NPs. In addition, utilizing stimuli-responsive in situ self-assembly or release in tumor microenvironment to design smart trackers and/or probes can also increase contrast signal, tracing veracity and guide-monitoring real-time performance of the targets. Meanwhile, some trackers holding capacity of ROS generation, drug release or heat production under light irradiation can realize precise and efficient treatment. Such trackers have attracted broad attention owing to the integration of diagnosis and therapy in a single formulation. Thus, for modern and even future theranostics in particular of stem-cell therapy and immunotherapy, the *in vivo* cell tracking combining multimodal technologies with integrated smart probes will be of great potentials.

## Figures and Tables

**Table 1:** Progress of biomedical nanoparticles (NPs) in the clinical trials.*^a^*

Study title / Diseases / Used nanoparticles	Progress / Locations
Pre-operative staging of pancreatic cancer using superparamagnetic iron oxide magnetic resonance imaging (SPIO MRI) / Pancreatic cancer / SPIONPs	Phase 4 (Completed)*^b^* / Massachusetts, US
Comparison of central venous catheters with silver nanoparticles versus conventional catheters central venous / Catheter related infections / Silver NPs (AgTive^®^)	Phase 4 (Completed) / ICU Rome, Italy
Clinical study on the harvesting lymph nodes with carbon nanoparticles for advanced gastric cancer / Advanced gastric cancer / Carbon NPs	Phase 3 (Unknown)*^c^* / Beijing, China
Multi-modality imaging (PCa) / prostate cancer, adenocarcinoma and neoplasm / [F-18]-DCFPyL injection (PET/MRI)	Phase 2 (Not recruiting)*^d^* / Ontario, Canada
Topical fluorescent nanoparticles conjugated somatostatin analog for suppression and bioimaging breast cancer / Breast cancer, skin cancer and diseases / CdS/ZnS core-shell type quantum dots coated with veldoreotide	Phase 1 (Recruiting)*^e^* / Qassim, Saudi Arabia

*^a^* This information is acquired from https://clinicaltrials.gov. *^b^* Completed: The study has ended normally, and participants are no longer being examined or treated. *^c^* Unknown: The study has passed its completion date, and the status has not been last verified within the past 2 years. *^d^* Not recruiting: The study has not started recruiting participants. *^e^* Recruiting: The study is currently recruiting participants.

**Table 2:** Trackers, advantages and disadvantages of various imaging techniques.

Techniques	Trackers / Advantages / Disadvantages
MRI, MPI	Trackers: Gd^3+^-, Mn^2+^-, ^19^F-based NPs, IONs and SPIONs. Advantages: (1) Arbitrary orientation tomography and non-osseous artifacts for accurate diagnosis; (2) Near-ideal depth penetration, spatial resolution (10~100 μm), high contrast and quantitative analysis; (3) Several weeks to months tracking for *in vivo* fate and aging of stem cells; (4) Non-ionizing and low frequency magnetic fields in MPI. Disadvantages: (1) Long scanning time and slow imaging speed; (2) Very insensitive to tumor calcification and *in vivo* calculus.
Fluorescence imaging	Trackers: inorganic QDs-, FNDs- and organic material-based NPs. Advantages: (1) Easy operation, low cost, non-invasive and fast imaging from seconds to minutes; (2) Relatively good biocompatibility for FNDs and organic material-based NPs; (3) High photostability for SP-based NPs and AIE dots; (4) Several weeks tracking for *in vivo* studies; (5) Theranostic capability based on some luminogens owning ROS generation. Disadvantages: (1) Limited tissue penetration depth in several micrometers for traditional optical probes, except for NIR-II probes (several millimeters); (2) Interference from biological auto-fluorescence; (3) Difficult to quantitative analysis.
PET/SPECT imaging	Trackers: radioisotopes-based materials such as ^18^F, ^64^Cu, ^89^Zr, ^111^In and ^86^Y/^177^Lu-based reagents. Advantages: (1) Non-invasive inspection for early stage cancers and further precise medication with high quality; (2) Unlimited detection depth, spatial resolution (1~2 mm) and quantitative analysis; (3) Short tracking period depending on the half-life of radioactive reagents; (4) Whole-body imaging with high sensitivity. Disadvantages: (1) High cost; (2) Use of radioisotopes leading to hard-to-assess *in vivo* security; (3) Low sensitive to tumors in lung, liver and gastrointestinal tract in comparison with MRI or CT.
PA imaging	Trackers: Au nanorods, graphene, carbon nanotubes, organic smell-molecules and SPNs etc. Advantages: (1) Non-invasive and non-ionizing detection and fast imaging from seconds to minutes; (2) Deep tissue penetration depth in centimeters with good SNR and spatial resolution in tens to hundred micrometers, rich contrast and high sensitivity; (3) Days to weeks tracking for *in vivo* fate and aging of stem cells; (4) Low cost and portable system. Disadvantages: (1) Limited imaging resolution in comparison with optical imaging; (2) Lack of stability in detecting methods, and shallow detection depth in comparison with mature clinic detection technology such as CT and MRI.

## Abbreviations

AIE: aggregation-induced emission
ACQ: aggregation caused quenching
AuNRs: gold nanorods
BBB: blood-brain barrier
Bro: bromelain
BSA: bovine serum albumin
cRGD: cyclo(Arg-Gly-Asp-D-Phe-Lys(mpa))
CAR T cell: chimeric antigen receptor T cell
CFSE: carboxyfluorescein diacetate succinimidyl ester
CLSM: confocal laser scanning microscope
CSCs: cancer stem cells
CT: computed tomography
D-A: donor-acceptor
DAPI: 4ʹ,6-diamidino-2-phenylindole
DCF-DA: 2ʹ,7ʹ-dichlorofluorescin diacetate
DLS: dynamic laser scattering
DMT1: divalent metal transporter 1
ECSCs: embryonal carcinoma stem cells
EdU: 5-ethynyl-2ʹ-deoxyuridine
EPR: enhanced permeability and retention
FDG: fluorodeoxyglucose
FNDs: fluorescent nanodiamonds
FLIM: fluorescence lifetime imaging microscopy
GAGs: glycosaminoglycans
hMSCs: human mesenchymal stem cells
LSCs: lung stem cells
MASIs: matrix-associated stem cell implants
mMSCs: marrow derived mesenchymal stem cells
MSCs: mesenchymal stem cells
IONs: iron oxide nanoparticles
MPI: magnetic particle imaging
MRI: magnetic resonance imaging
Mn: manganese
NV^-^: nitrogen-vacancy
NIR: near-infrared
NIR-I: first near-infrared
NIR-II: second near-infrared
NIS: sodium iodide symporter
NPs: nanoparticles
ODMR: optically detected magnetic resonance
OR-PAM: optical-resolution photoacoustic microscopy
PAA: polyacrylic acid
PA: photoacoustic
PDT: photodynamic therapy
PET: positron emission tomography
PSs: photosensitizers
PTT: photothermal therapy
QDs: quantum dots
QY: quantum yield
ROS: reactive oxygen species
SBR: signal-to-background ratio
SDT: sonodynamic therapy
SMI: small molecule kinase inhibitor
SNR: signal-to-noise ratio
SPECT: single photon emission computing tomography
SPIONs: superparamagnetic iron oxide nanoparticles
SPs: semiconducting polymers
SPNs: semiconducting polymer nanoparticle
T/NT ratio: tumor/normal tissue signal ratio
TEM: transmission electron microscope
TGF: transforming growth factor
US: ultrasound
USPIONs: ultrasmall iron oxide nanoparticles
VacV: vaccinia virus
